# Systematics and Evolution of the Miocene Three-Horned Palaeomerycid Ruminants (Mammalia, Cetartiodactyla)

**DOI:** 10.1371/journal.pone.0143034

**Published:** 2015-12-02

**Authors:** Israel M. Sánchez, Juan L. Cantalapiedra, María Ríos, Victoria Quiralte, Jorge Morales

**Affiliations:** 1 Departamento de Paleobiología, Museo Nacional de Ciencias Naturales-CSIC, Madrid, Spain; 2 Leibniz-Institut für Evolutions und Biodiversitätsforschung, Museum für Naturkunde, Berlin, Germany; NYIT College of Osteopathic Medicine, UNITED STATES

## Abstract

Palaeomerycids were strange three-horned Eurasian Miocene ruminants known through fossils from Spain to China. We here study their systematics, offering the first cladistic phylogeny of the best-known species of the group, and also reassess their phylogenetic position among ruminants, which is currently disputed. The beautifully preserved remains of a new palaeomerycid from middle Miocene deposits of Spain, *Xenokeryx amidalae* gen. et sp. nov., helps us to better understand palaeomerycid anatomy, especially that of the nuchal region in the skull, significantly improving our current knowledge on these enigmatic ruminants. Our results show two main lineages of palaeomerycids, one containing the genus *Ampelomeryx* diagnosed by a characteristic type of cranium / cranial appendages and some dental derived traits, and another one that clusters those forms more closely related to *Triceromeryx* than to *Ampelomeryx*, characterized by a more derived dentition and a set of apomorphic cranial features. *Xenokeryx* branches as a basal offshoot of this clade. Also, we find that Eurasian palaeomerycids are not closely related to North American dromomerycids, thus rejecting the currently more accepted view of palaeomerycids as the Eurasian part of the dromomerycid lineage. Instead of this, palaeomerycids are nested with the African Miocene pecoran *Propalaeoryx* and with giraffoids. On the other hand, dromomerycids are closely related to cervids. We define a clade Giraffomorpha that includes palaeomerycids and giraffids, and propose an emended diagnosis of the Palaeomerycidae based on cranial and postcranial characters, including several features of the cranium not described so far. We also define the Palaeomerycidae as the least inclusive clade of pecorans containing *Triceromeryx* and *Ampelomeryx*. Finally, we reassess the taxonomy of several palaeomerycid taxa.

## Introduction

Ruminants are possibly the most successful group of herbivore mammals both in terms of diversity and biomass (extinct and extant), and also the most diverse of extant terrestrial cetartiodactyls (the clade of mammals containing ruminants, hippos, cetaceans, camels, peccaries and pigs). They appeared in the late Eocene producing several basal lineages that became successive sister groups to the clade Pecora (the more derived ruminants). Out of the six extant ruminant families, five are pecorans (musk-deer, pronghorns, cervids, bovids and giraffes) whereas tragulids (chevrotains and mouse-deer) are relics of the ancient non-pecoran groups. Pecorans flourished during the Miocene (between 24 and 5 Ma), experiencing radiations that gave rise to the modern lineages, and spreading throughout Eurasia, Africa and North America. One of the most amazing evolutionary novelties of pecorans is the development of cranial appendages in several extinct and extant families [1]. These cranial structures are of two basic types attending to their origin: apophyseal (i.e. out-growths of the skull) and epiphyseal (i.e. developed apart from the skull and later fused to it) [1–3].

The Paleomerycidae comprised a group of strange-looking pecorans that inhabited Eurasia from the late early to the late Miocene [4–7]. Some claims have been made of African palaeomerycids [8–10], but all these remains were later re-interpreted as belonging either to giraffoid climacoceratids [11] or female individuals of the bizarre pecoran *Prolibytherium* [12]. Palaeomerycids displayed a pair of unbranched non-deciduous epiphyseal frontal appendages (ossicones) similar to those of giraffids that were cylindrical to flattish in cross-section. They also had a forked supra-occipital appendage of apophyseal origin that was variable both in morphology and size among the different taxa [4,6,10,13,14]. The occipital appendage of the Chinese ‘*Palaeomeryx’ tricornis* was originally described as a ‘bony horn much dilated at its end’ [15], and reconstructed as a non-forked structure. However more recent discoveries have demonstrated that the occipital appendage of ‘*P*.’ *tricornis* was in fact long and bifurcated [16]. Palaeomerycid females were apparently hornless and the males sported large sabre-like upper canines [13,15].

The first remains of palaeomerycids were originally described on the basis of middle Miocene fossils from Georgensmünd, Germany [17]. Subsequently, palaeomerycid fossils have been found in other parts of Europe [7,13,14,18,19] and China [15,20]. The group is particularly well-known from the middle Miocene of the Iberian Peninsula, displaying a good diversity of forms [13,21–24]. Some of these Spanish remains are among the best palaeomerycid samples described (e.g. the discovery of *Triceromeryx pachecoi* Villalta et al., 1946 showed for the first time the full array of cranial appendages in the Palaeomerycidae). Along with *Palaeomeryx*, five more genera have been currently described: *Triceromeryx*, *Ampelomeryx* and *Tauromeryx* in Spain, with some scarce Chinese remains ascribed to *Triceromeryx* by Bohlin [20], *Germanomeryx* in Germany [6,13,21,24] and *Sinomeryx* [13] for the Chinese form previously published by Qiu et al. [15] as *Palaeomeryx tricornis*. Classically, the diagnosis and definition of the Palaeomerycidae have been highly variable. Rössner [6] offers a very complete resume of all these systematic issues. Starting with Lydekker [25], which erected the family name Palaeomerycidae, some authors diagnosed palaeomerycids on the basis of dental features and included within the group hornless forms such as *Amphitragulus* or *Oriomeryx*, which in turn were considered moschids in some other publications and some of them finally turned out to be basal pecorans [26]. On the other hand, some authors diagnosed palaeomerycids by the presence of ossicones and a single occipital appendage, and considered a more restricted group [4,7,13,19,27–29]. *Prolibytherium* was sometimes considered part of the Palaeomerycidae despite its tremendous differences with the three-horned true palaeomerycids [10,13], however later works have assigned this taxon to Giraffoidea and Climacoceratidae [26,30]. As noted by Rössner [6] the suite of dental characters used by Janis and Scott [27] to diagnose the family Palaeomerycidae is present in other taxa such as cervids or moschids, and the only real autapomorphic features of palaeomerycids recognized so far are the presence of both a bifurcated occipital appendage and a pair of supra-orbital ossicones.

The phylogenetic affinities of palaeomerycids within the Pecora have also been subject of great dispute. Early authors such as Scott [31] and Stirton [32] suggested a close relationship with the North American Dromomerycidae, another group of deer-sized pecorans that had some three-horned representatives [5]. This point of view has been repeatedly followed by a considerable number of authors [5,10,22,27–29,33–35]. Among these works, the putative dromomerycid-palaeomerycid lineage was in turn variably related with the Cervidae or the Giraffidae. For example, [28] makes palaeomerycids and dromomerycids sister groups and links them with giraffids and bovids. However, this ‘Dromomerycinae-Palaeomerycinae’ hypothesis was contested [4,7,13] arguing that the frontal ossicones of palaeomerycids were apparently distinct from the frontal appendages of dromomerycids, which never show the basal suture typical of ossicones and appear to have an apophyseal origin [1]. Also, the occipital appendage of dromomerycids, when present, is simple instead of forked. Our personal examination of dromomerycid material curated by the American Museum of Natural History (New York) revealed big differences with palaeomerycids in the morphological construction of the occipital appendage, the nuchal plane, the external morphology of the frontal appendages, and in several key postcranial characters. Hence, as pointed out by Duranthon et al. [13] among others, the hypothesis of a close relationship between the two groups can be severely questioned. Apart from the possible direct relationship with dromomerycids, palaeomerycids have been alternatively placed close to giraffids [9,10,36] and cervids [4,13,27,29], a question that also remained unsolved.

**Fig 1:**
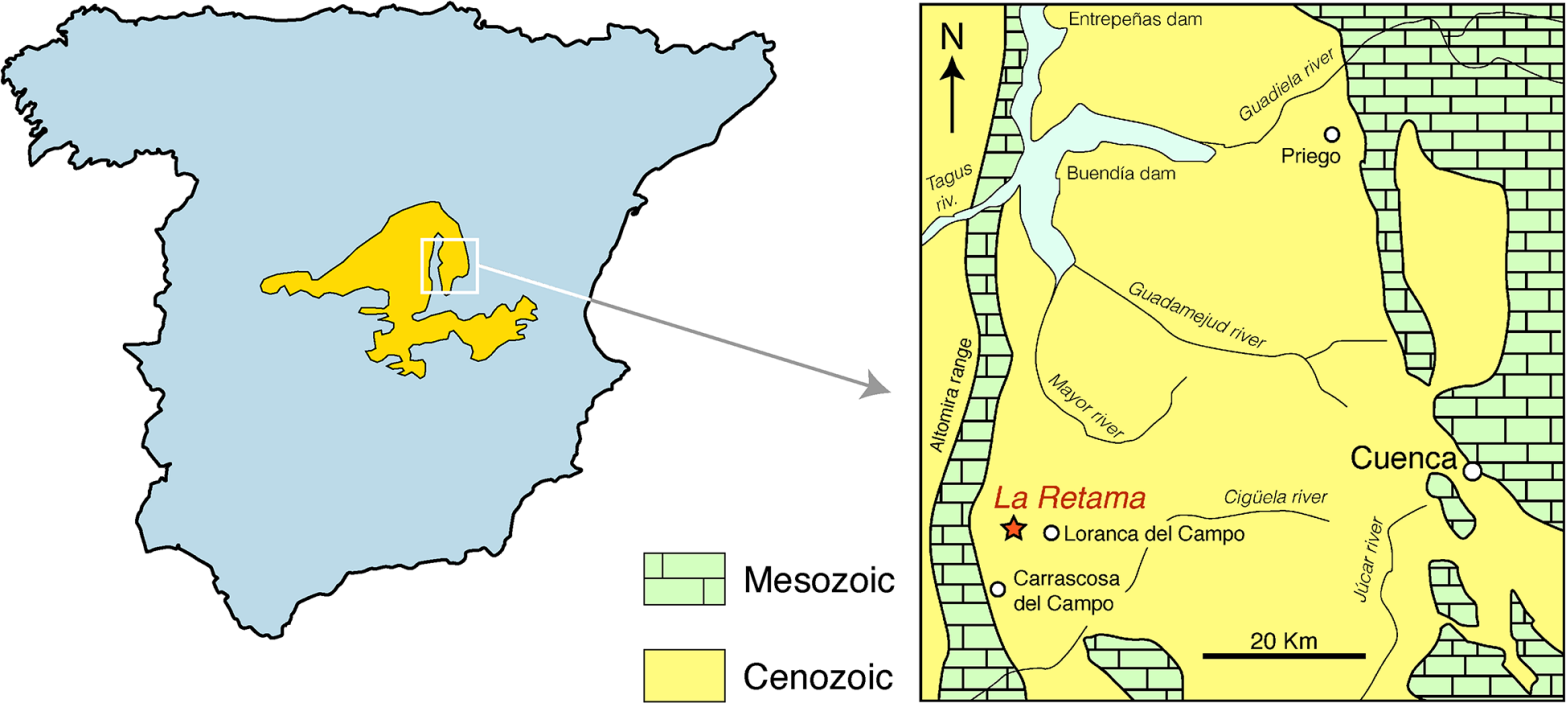
Geological and geographical setting of La Retama fossil site within the Loranca Basin in the Iberian Peninsula. Map illustration by IMS, modified from [41].

In this paper we describe the remains of a new palaeomerycid from the middle Miocene (MN5) fossil site of La Retama (Loranca Basin, Cuenca province, Spain; Fig 1), represented by a complete sample of cranial (including both frontal and supra-occipital cranial appendages), dental and postcranial remains which are relevant to study the systematics and evolution of the Paleomerycidae. The aims of this work are: a) to describe these new fossils and explore for the first time the phylogenetic relationships among the better known forms of palaeomerycids to understand their evolution and reassess their systematics; b) to test the hypothesis of palaeomerycids and dromomerycids not being closely related, exploring their phylogenetic relationships within the Pecora; and c) to achieve a good diagnosis and definition of the clade Palaeomerycidae.

## Locality and Geological Setting

The fossil site of La Retama (40°5’9.03”N, 2°44’29.22”W; Fig 1) is located in the Loranca basin, in the crop fields that extend near the town of Loranca del Campo (Cuenca province, Spain). The Loranca basin is a long and narrow marginal depression with a N-S oriented main axis. The stratigraphy of the Loranca basin has been thoroughly described in a number of publications [37–40]. The site was discovered in august 1989 during the field campaign in the nearby sites of Loranca (lower Miocene; [41]). The fossiliferous levels correspond with massive marly clays and deltaic facies [41] with carbonate concretions of diagenetic origin. La Retama preserves an abundant fossil fauna that includes gastropods and vertebrates such as chelonians, crocodiles, lagomorphs, rodents, several carnivorans, anchitheriine equids, rhinoceroses, gomphoteriid elephants, caenotheriids, suids, cervids and palaeomerycids [41,42]. However, cervid remains are very scarce. Oddly enough, the basal bovid *Eotragus*, which is known from other Spanish sites of equivalent age, is absent from La Retama. There is a strong predominance of *Anchitherium castellanum*, with a moderate abundance of rhinoceroses and palaeomerycids. Among micromammals the ground squirrel *Heteroxerus* is the most abundant. The paleoenvironment of La Retama was interpreted as an open area with more or less permanent water bodies and a warm seasonal climate [41]. The estimated age for La Retama is ca. 15.4–15.9 Ma (local zone Db, MN5; [43]).

## Materials and Methods

### Material

The new palaeomerycid described in this work is based upon the complete sample of non-articulated palaeomerycid material from La Retama curated by the MNCN-CSIC (Madrid, Spain). *Triceromeryx pachecoi* data come from the original specimens from La Hidroeléctrica (Madrid) curated by the MNCN-CSIC and first described by Villalta et al. [21]. *Ampelomeryx ginsburgi* data come from casts stored at the MNCN-CSIC and original fossils curated by the ICP (Barcelona, Spain). *Tauromeryx turiasonensis* data come from casts stored at the MNCN-CSIC and original material curated by the Paleontology Museum of the University of Zaragoza (Zaragoza, Spain). The unnamed form from Mesegar-2 (Toledo Province, Spain) is curated by the MNCN-CSIC. Morphological data of *Palaeomeryx tricornis*, *Palaeomeryx kaupi*, *Palaeomeryx magnus* and *Germanomeryx fahlbuschi* come from their respective original publications and / or recent revisions and photographs of the original material [6, 7, 15, 17]. Data of *Cranioceras*, *Sinclairomeryx*, *Merycodus*, *Stockoceros* and *Antilocapra* come from original material curated by the AMNH (New York, USA), with an additional *Antilocapra* adult male specimen (skull only) stored at the MNCN-CSIC. *Moschus* data come from specimens curated by the AMNH (New York, USA), the Museum of Zoology of the University of Cambridge (Cambridge, UK), and the Museo Anatómico de la Universidad de Valladolid (Valladolid, Spain). Fossil moschids comprise material published and cited in [26,44–46]. Cervid data were taken from osteological material of extant *Muntiacus* curated by the Museo Anatómico de la Universidad de Valladolid (Valladolid, Spain) and the MNCN-CSIC, and extant *Capreolus* curated by the MNCN-CSIC. Data regarding giraffids and tragulids come from the collections of comparative anatomy of the MNCN-CSIC, the AMNH (New York, USA) and the Museum of Zoology of the University of Cambridge (Cambridge, UK). Data of *Dremotherium* come from the type locality of Saint-Gérand-le-Puy, France, curated by the MNHN (Paris, France) and from the fossil sample of Cetina de Aragón, Spain ([47]; Sánchez pers. obs.), curated by the MNCN-CSIC (Madrid, Spain). Data of *Amphitragulus* come from the French localities of Saint-Gérand-le-Puy and Quercy, curated by the MNHN (Paris, France). Data of *Gelocus communis* come from casts of the type material stored at the MNCN-CSIC. Data regarding *Orangemeryx*, *Namibiomeryx*, *Propalaeoryx* and *Namacerus* come from the original material from the Sperrgebiet, Namibia [11,41,48–50]. Data of *Eudorcas thomsonii* come from osteological material stored at the MNCN-CSIC. Finally, several morphological data come from Janis and Scott [27] and Webb and Taylor [51].

The mitochondrial genomes of *Hyemoschus*, *Muntiacus*, *Capreolus*, *Moschus*, *Eudorcas*, *Giraffa* and *Antilocapra* are part of the original dataset presented by Hassanin et al. [52] and were downloaded from GenBank (accession numbers NC_020714, FJ705435, JN632662, JN632645 and JN632597 respectively).

No permits were required for the described study of La Retama fossils, which complied with all relevant regulations.

### Measurements

All measurements are presented in S1 Table and S1 Text and were taken with digital calipers. We follow the set of measurements proposed by Quiralte [53].

### Nomenclature

We use the terminology of Barone [54] for anatomic nomenclature of the cranial and postcranial skeleton, and that published by Azanza [55] and Sánchez and Morales [44] for nomenclature of the dentition.

### Phylogenetic analysis

Despite the existence of reasonably good anatomical information, a reconstruction of the phylogenetic relationships between the different palaeomerycid forms has not been attempted so far. Here we present the first phylogenetic reconstruction of the group. We chose the early Miocene African pecoran *Propalaeoryx* as the outgroup due to its close relationship with the palaeomerycid clade (see the pecoran trees in this work). The ingroup is composed by several previously published palaeomerycids: *Triceromeryx pachecoi*, ‘*Palaeomeryx*’ *magnus* (Sansan), *Tauromeryx turiasonensis*, *Palaeomeryx kaupi* (Georgensmünd), *Ampelomeryx ginsburgi*, ‘*Sinomeryx*’ *tricornis* and ‘*Germanomeryx*’ *fahlbuschi*. In addition we included the new palaeomerycid from La Retama (*Xenokeryx amidalae*) and an unnamed and not yet described form from the Spanish site of Mesegar-2 (MN4, Tagus Basin, Toledo Province) that added useful information to the morphological dataset. Also, a second batch of phylogenetic analyses (MP-morphology and Bayesian-combined DNA + morphology) were performed to explore the position of the Palaeomerycidae within the Pecora and test the hypothesis of palaeomerycids and dromomerycids not being closely related. We chose the extant African chevrotain *Hyemoschus* as the outgroup following Sánchez et al. [26,45]. The ingroup included basal pecorans such as *Gelocus* and *Amphitragulus*, three palaeomerycids (*Xenokeryx*, *Triceromeryx* and *Ampelomeryx*), *Prolibytherium*, the climacoceratid *Orangemeryx*, the African pecoran *Propalaeoryx*, the extant giraffid *Giraffa*, the hornless pecorans *Namibiomeryx*, *Blastomeryx* and *Dremotherium*, two dromomerycids pertaining to the two described dromomerycid clades (*Cranioceras* and *Sinclairomeryx*; [34]), the extant cervids *Capreolus* and *Muntiacus*, the stem bovoid *Sperrgebietomeryx* [26], the merycodontid *Merycodus* and two antilocaprids (*Stockoceros* and extant *Antilocapra*), the moschids *Hispanomeryx*, *Micromeryx*, *Moschus* and ‘*Moschus’ grandeavus*, the basal bovid *Namacerus* and the extant bovid *Eudorcas*.

The data matrices were compiled in MacClade 4.05 and transformed using Mesquite 3.01 (Macintosh versions).

#### Maximum Parsimony analysis

We run a Maximum Parsimony analysis for checking the position of the palaeomerycid from La Retama within the Palaeomerycidae, exploring the phylogenetic frame of the group. We used a morphological dataset of 32 characters (cranial, dental and postcranial) with 10 OTUs including the outgroup. Also, we made an additional MP analysis to test the hypothesis of relationship of palaeomerycids within the Pecora, using a modified morphological dataset from Sánchez et al. [45]. This dataset includes 67 characters (cranial, dental and postcranial) and 27 OTUs including the outgroup. We used TNT v1.1. software [56] to analyze both datasets. In both cases all characters are non-additive and unweighted, and the trees were searched using a Traditional Search method (heuristic algorithm) with TBR and 1000 replicates (holding 10 most parsimonious trees for each replicate). Bootstrap (1000 replicates) was used as branch support assessment.

#### Bayesian tip-dating analysis

In addition to our maximum parsimony approach, we performed a ‘tip-dating’ Bayesian analysis [57] with the same 27 OTUs. Likelihood-based phylogenetic inference has been acknowledged to be less sensitive to homoplasy than traditional parsimony, which treats fast-evolving (homoplasic) and conservative characters in the same way [58]. Additionally, the ‘tip-dating’ method provides the utility of a simultaneous estimation of tree topology and divergence times based on a relaxed morphological and/or molecular clocks and the stratigraphic range of the fossil taxa (used for non-contemporaneous sampling) [57,59]. One of the advantages of this method is that morphological and molecular data can be combined and modeled separately to infer a timetree. To increment the power of the phylogenetic estimates (both in terms of topology and branching times), we complemented the morphological dataset (the same used for Parsimony analysis) with mitochondrial DNA for the seven extant genera included in the morphological matrix (*Hyemoschus*, *Muntiacus*, *Capreolus*, *Moschus*, *Eudorcas*, *Giraffa* and *Antilocapra*). Mitochondrial sequences were initially aligned using MAFFT [60] and revised using Mesquite [61]. In particular, our analyses were performed using 4 molecular partitions: 12S (970bp), 16S (1560), COX3 (784) and CytB (1125), representing a total of 4439 bp. We used the R package BEASTMaster (phylo.wikidot.com/beastmaster) [62] for combining the morphological and molecular datasets and translate them into a BEAST XML file. BEASTMaster provides BEAST2 [63] with congruent birth-death tree as well as relaxed morphological and molecular models. Our analysis used a BDSS (birth-death with serial sampling, disallowing direct ancestors) tree prior. *Hyemoschus* was set as the outgroup and the root age prior used was a normal distribution between 41 and 29 Ma. These limits were established by combining the 95% ranges of crown Ruminantia from two recent molecular estimates that used informed fossil-derived node constrains [64,65]. *Archeomeryx*, the putative oldest and most basal ruminant, has a temporal range that may span up to ~48 Ma. However, the phylogenetic position of this taxon is not clear [66]. We used uniform priors for fossil tip dates based on the corresponding stratigraphic ranges. The analysis was run twice for 20 million generations, sampling every 1000^th^ generation. We used Tracer v 1.6 [67] to evaluate both chains reaching stationary, the effective sample sizes were above 200 for all parameters, and both runs yielded convergent results. We used LogCombiner v2.1.3 in order to generate a combined tree file from both runs and discard the burning (10% of each run). The maximum credibility tree was obtained using TreeAnnotator v2.1.2 [68] and median divergence dates recorded to the summary tree.

Both the data matrices for all the analyses and the lists of characters are presented in S2 and S3 Text, and S1–S3 Files.

### Nomenclatural acts

The electronic edition of this article conforms to the requirements of the amended International Code of Zoological Nomenclature, and hence the new names contained herein are available under that Code from the electronic edition of this article. This published work and the nomenclatural acts it contains have been registered in ZooBank, the online registration system for the ICZN. The ZooBank LSIDs (Life Science Identifiers) can be resolved and the associated information viewed through any standard web browser by appending the LSID to the prefix “http://zoobank.org/”. The LSID for this publication is: urn:lsid:zoobank.org:pub:CB41B04D-8AE6-4AD8-A74B-9F15901376F6. The electronic edition of this work was published in a journal with an ISSN, and has been archived and is available from the following digital repositories: PubMed Central, LOCKSS.

## Systematic Palaeontology

MAMMALIA Linnaeus, 1758
CETARTIODACTYLA Montgelard, Catzeflis and Douzery, 1997
RUMINANTIA Scopoli, 1777
PECORA sensu Webb and Taylor, 1980
PALAEOMERYCIDAE Lydekker, 1883

### Emended diagnosis of the Palaeomerycidae

Pecorans with the following synapomorphic combination: presence of frontal (supra-orbital) ossicones and a single, branched, occipital appendage that involve the elongation and modification of the nuchal plane and the supra-occipital; presence of nuchal fossa; presence of a laterally-oriented expansion of the nuchal crest; presence of a well-developed crest in the proximo-plantomedial process of the navicular cuboid that does not reach the proximal region of the process.

### Genus *Palaeomeryx* von Meyer, 1834 [17]

*Palaeomeryx* was described on the basis of several teeth and scarce skeletal remains from the Miocene locality of Georgensmünd, with the type species *P*. *kaupi* [17]. The cranial appendages of this form are unknown. As noted by Duranthon et al. [13], palaeomerycids show a great diversity in the morphology of the cranial appendages but maintain a homogeneously plesiomorphic dentition, being the cranial appendages a key feature for the taxonomy and systematics of the group. Hence, as Astibia [7] pointed out and we confirm in this work, the material from Georgensmünd does not appear to be diagnostic. For these reasons we follow Duranthon et al. [13], Rössner [6], and Astibia [7] in regarding this form as *species inquirenda* and restricting the genus name *Palaeomeryx* to the Georgensmünd remains described by von Meyer [17]. Included species: *Palaeomeryx kaupi* von Meyer, 1834.

### Genus *Ampelomeryx* Duranthon et al., 1995 [13]

#### Emended diagnosis

Flattish and not pneumatized ossicones with forward-oriented extension ‘wing’; presence of ‘eyebrow’ supraorbital projections basal to the ossicones; nuchal crest extended into the shaft of the occipital appendage; sloped occipital appendage of variable length (depending of the species) oriented in an open angle with respect to the parietals [13] and with rounded tips; lack of longitudinal crests in the posterior face of the occipital appendage; elongated and large nuchal extension; well-developed *Palaeomeryx*-fold; elongated and buccally placed hypoconulid in the m3. Included species: *Ampelomeryx ginsburgi* Duranthon et al., 1995; *Ampelomeryx tricornis* (Qiu et al., 1985), comb. nov.; *Ampelomeryx fahlbuschi* (Rössner, 2010), comb. nov.

### Genus *Triceromeryx* Villalta et al., 1946 [21]

#### Emended diagnosis

Y-shaped and broad occipital appendage with well-developed pedicle and cylindrical branches; well-marked and triangular posterior groove in the occipital appendage, with the apex pointing upwards; very well-developed posterior longitudinal ‘rods’ in the occipital appendage; ossicones with large and individualized bumps, more or less abundant and concentrated in the posterior face of the ossicone; buccally-oriented third lobe in the m3; cranio-caudally developed proximo-lateral tubercle in the radius. Included species: *Triceromeryx pachecoi* Villalta et al., 1946; *Triceromeryx tsaidamensis* Bohlin, 1953; *Triceromeryx magnus* (Lartet, 1851), comb. nov.

### Genus *Tauromeryx* Astibia et al., 1998 [24]

#### Emended diagnosis

Long, pointed and smooth ossicones with no bumps and absent extension ‘wing’; sloped Y-shaped and narrow occipital appendage with small conical branches and absent or nearly absent pedicle; winged buccal cone in the P4; straight distolateral border of the distal trochlea in the astragalus, showing no notch. Included species: *Tauromeryx turiasonensis* (Astibia and Morales, 1987).

### Genus *Xenokeryx* nov.

urn:lsid:zoobank.org:act:BF7F79F9-6752-4CD2-8022-64C5F5790D57

#### Etymology

*Xenos*, greek for strange, *keryx* referring to horn. Meaning ‘strange horn’.

#### Diagnosis

T-shaped upright occipital appendage with well-developed pedicle and downwards-oriented branch tips; very faint longitudinal crests in the posterior face of the occipital appendage; ulna distally fused to radius; short palmar extension of the facet for the semilunate in the radius; straight disto-lateral border of the distal trochlea in the astragalus, showing no notch; distal articulation facet of the first phalanx not extended into the flexor area.

### *Xenokeryx amidalae* sp. nov.

urn:lsid:zoobank.org:act:9B119A4F-AB1F-4077-A6F6-31981F294A64

#### Synonyms

*Triceromeryx conquensis*, nomen nudum (in ref. [69], p. 63, 88); *Triceromeryx conquensis*, nomen nudum (in ref. [70], p. 117); *Triceromeryx* sp. nov. (in ref. [41], p. 257)

#### Etymology

Referred to the fictional character Padme Amidala from Star Wars, due to the striking resemblance that the occipital appendage of *Xenokeryx* bears to one of the hairstyles that the aforementioned character shows in The Phantom Menace feature film.

#### Diagnosis

The same as the genus.

#### Holotype

MNCN-74448, complete occipital appendage of an adult individual.

#### Paratypes

The remaining referred material from La Retama.

#### Locality, age and horizon

La Retama, middle Miocene, middle Aragonian, MN5, local zone Db [43].

#### Type and only species

Xenokeryx amidalae gen. et sp. nov.

#### Material

MNCN-74458 (right hemimandible with p3-m3); MNCN-74456 (left mandibular fragment with m1-m3); MNCN-74455 (right mandibular fragment with m2-m3); MNCN-74495 (left maxillary fragment with DP2-M1); MNCN-74450 (left P4-M1); MNCN-74457 (right m3); MNCN-74453 (left P2); MNCN-74451 (right P3); MNCN-74452 (right M3); MNCN-74496 (left m1); MNCN-74454 (left P4); MNCN-74448 (holotype; occipital appendage); MNCN-74449 (right ossicone); MNCN-74446 (left juvenile ossicone); MNCN-74447 (left juvenile ossicone); MNCN-74486 (first phalanx); MNCN-74488 (distal fragment of first phalanx); MNCN-74487 (second phalanx); MNCN-74489 (second phalanx); MNCN-74494 (fragment of left pyramidal); MNCN-74493 (right pyramidal); MNCN-74491 (right pyramidal); MNCN-74484 (right semilunate); MNCN-74483 (left magnotrapezoid); MNCN-74482 (right malleolar); MNCN-74481 (left malleolar); MNCN-74499 (right navicular-cuboid); MNCN-74479 (left navicular-cuboid); MNCN-74480 (right navicular-cuboid); MNCN-74477 (right navicular-cuboid); MNCN-74490 (fragment of left navicular-cuboid); MNCN-74500 (right ectomesocuneiform); MNCN-74501 (right ectomesocuneiform); MNCN-74502 (right ectomesocuneiform); MNCN-74476 (left astragalus); MNCN-74470 (right calcaneus); MNCN-74469 (right calcaneus); MNCN-74461 (fragment of left scapula); MNCN-74473 (proximal fragment of left radius); MNCN-74460 (proximal fragment of right radius); MNCN-74459 (distal fragment of right radius); MNCN-74471 (proximal fragment of left femur); MNCN-74466 (distal fragment of left tibia); MNCN-74472 (proximal fragment of right metacarpal III-IV); MNCN-74474 (proximal fragment of metatarsal III-IV); MNCN-74464 (proximal fragment of left metatarsal III-IV with diaphysis); MNCN-74504, MNCN-74468, MNCN-74467 (distal metapodial trochlea); MNCN-74465 (distal fragment of metacarpal III-IV); MNCN-74475 (distal fragment of metatarsal III-IV).

## Description

### Cranial skeleton

**Fig 2:**
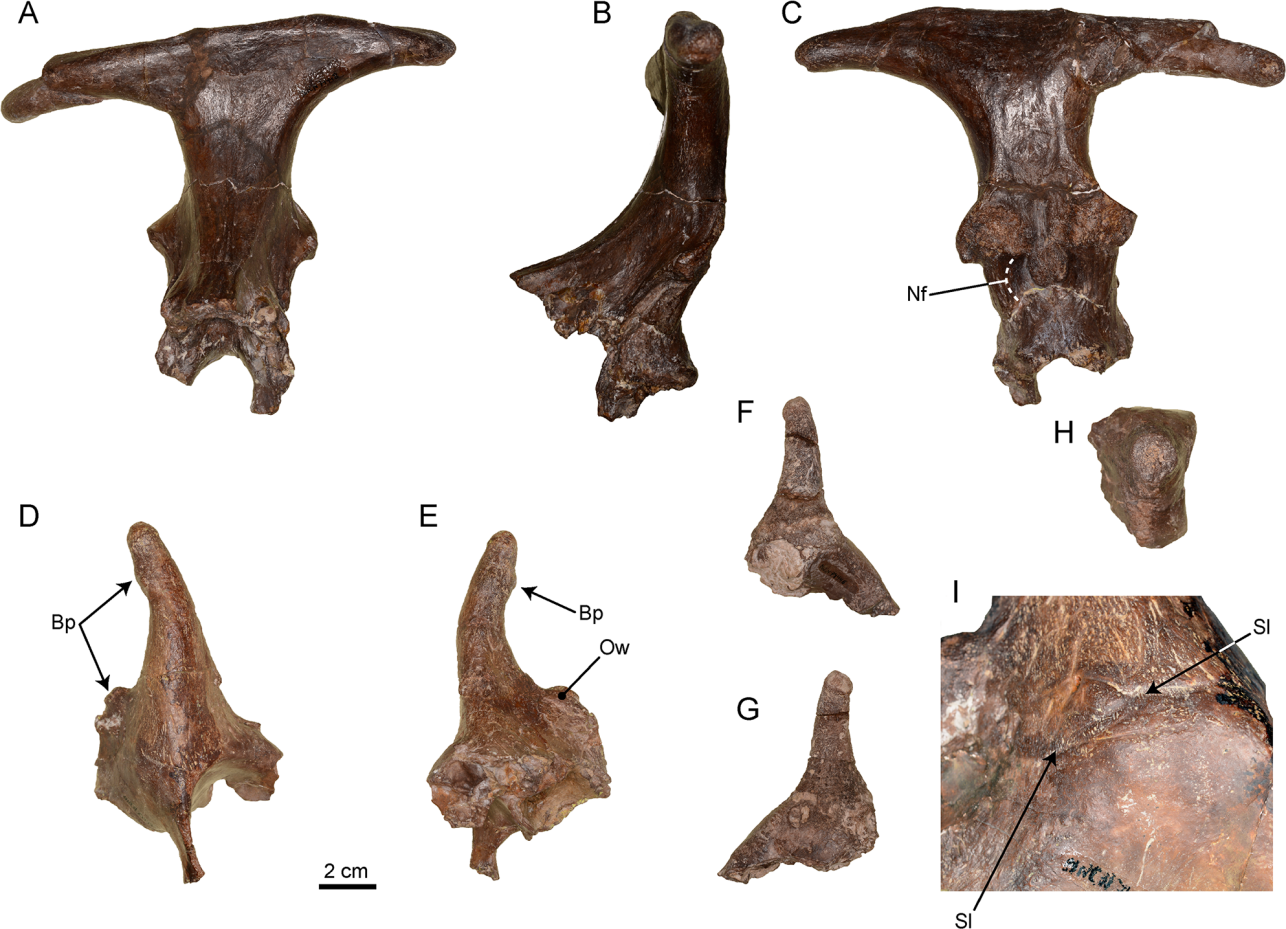
Cranial remains of *Xenokeryx amidalae* gen. et sp. nov. from La Retama. A, MNCN-74448 (holotype), occipital appendage in anterior view; B, MNCN-74448 (holotype), occipital appendage in lateral view; C, MNCN-74448 (holotype), occipital appendage in posterior view; D, MNCN-74449, right ossicone and supra-orbital region of a skull in latero-distal view; E, MNCN-74449, right ossicone and supra-orbital region of the skull in medial view; F, MNCN-74446, left juvenile ossicone and supra-orbital region of the skull in lateral view; G, MNCN-74446, left juvenile ossicone and supra-orbital region of the skull in medial view; H, MNCN-74447, left juvenile ossicone in apical view, showing its transversal section; I, MNCN-74449, detail of the ossicone-frontal bone contact showing the suture line (not to scale). Abbreviations: Bp, bumps; Nf, nuchal fossa; Ow, ossicone ‘wing’; Sl, suture line between ossicone and frontal bone.

The cranial remains (Fig 2) consist in supraorbital fragments and an occipital appendage with part of the posterior skull attached.

#### Ossicones

There are three preserved ossicones (Fig 2D–2I). The best specimen (MNCN-74449) is complete and has a good part of frontal bone attached, including the roof of the orbit and its posterior bar. MNCN-74446 and MNCN-74447 pertained very probably to immature specimens due to their porous surface and smaller size and also because of the thinner frontal bone attached. The ossicones of *Xenokeryx* are vertically arranged on the frontal bone, with apices oriented both backwards and inwards. They show a short extension ‘wing’ that is located at the back of the appendage and that sport a pair of isolated rounded bumps. The cross-section of the ossicones is subtriangular at their base and cylindrical from mid-shaft to the tip. The tip is rounded and wrinkled, very similar to that of the giraffes, and is surrounded by several bumps smaller than those present in the basal extension ‘wing’. The frontal bone is pneumatized at the base of the ossicones, and as observed in MNCN-74446 this pneumatization extends into the appendage, as occurs in *Triceromeryx* (but not in *Ampelomeryx*). The ossicone MNCN-74446 is transversally cut off just above the base. The cross-section is circular in shape, with a thick cortex of more dense bone and a far more porous core.

#### Occipital appendage

The specimen MNCN-74448 (holotype; Fig 2A–2C) is a beautifully preserved occipital appendage of an adult individual, including the complete nuchal plane and the supra-occipital area. There are no parietals and no mastoids preserved. The appendage is very robust with a well-developed upright pedicle. It develops into a large T-shaped terminal structure with downwards-oriented branch tips. The posterior surface of the appendage is smooth and slightly convex, lacking the well-marked rods present in *Triceromeryx* and the ridges of *Ampelomeryx*, having instead a couple of very faint longitudinal crests. The nuchal crest extends laterally into two triangular expansions that bear marks of muscular / tendinous attachment. A deep canal runs under these expansions, very similar to that present in the climacoceratid *Propalaeoryx*. The nuchal plane stretches out upwards enlarging the available surface for muscles and tendons and forming a central concave area over the foramen magnum region that we call herein the nuchal fossa. In this enlarged region the attachment areas are separated in at least two paired zones. The upper one extends over the aforementioned lateral expansions of the nuchal crest serving as probable attachment areas for the *rectus capitis dorsalis* and *semispinalis capitis* muscle packs. The lower elliptical attachment areas, located in the center of the nuchal fossa, are much smaller. They contact each other and probably served as attachment area for the *rectus capitis dorsalis minor* muscle. Both muscular sets originate in the upper edge of the spine in the second neck vertebra and are head extensors. On the upper side of the skull / appendage there is a wrinkled area, more exaggerated over the sagittal plane, that extends on the appendage like the very faint remains of a sagittal crest.

### Dentition

#### Upper dentition

**Fig 3:**
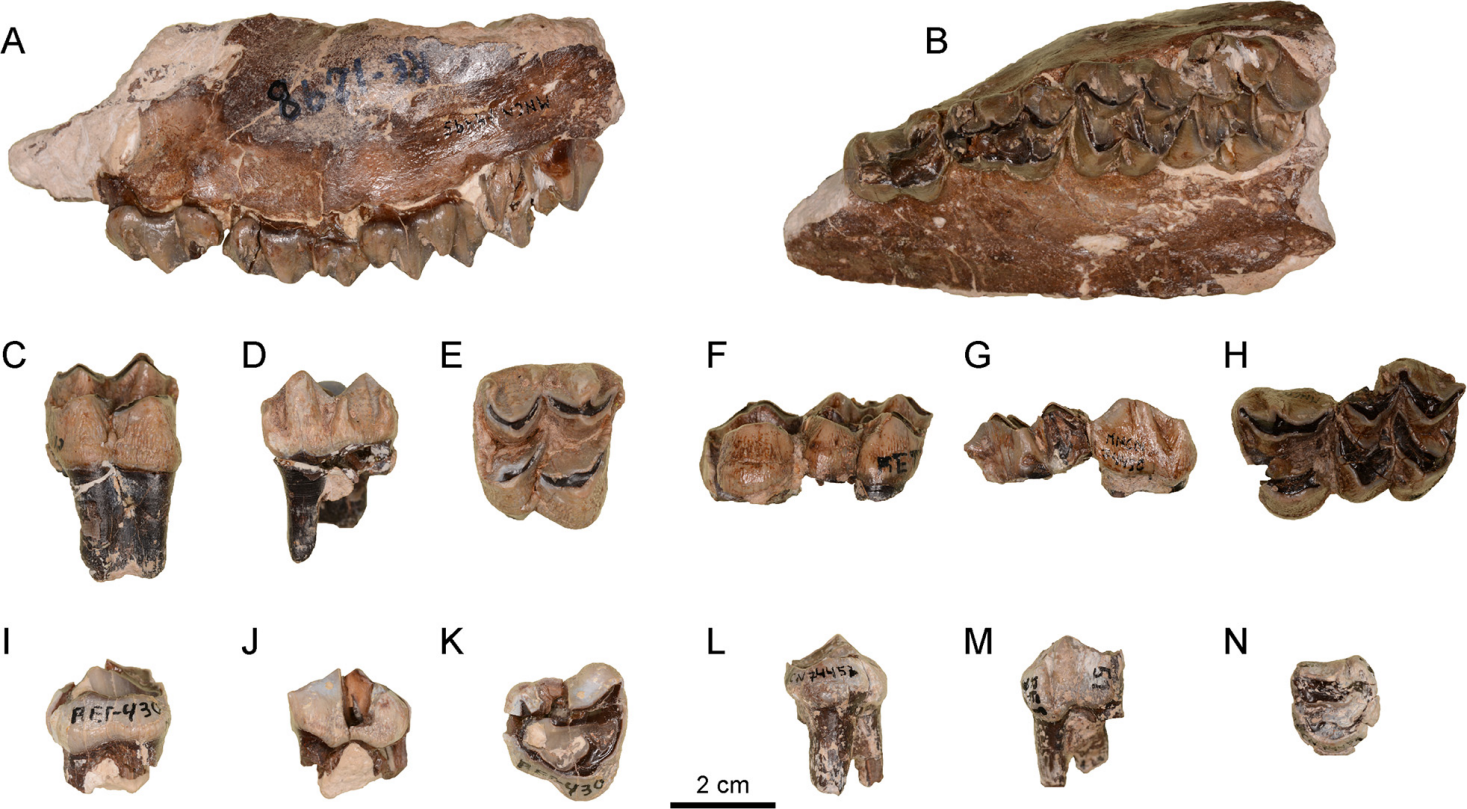
Upper dentition remains of *Xenokeryx amidalae* gen. et. sp. nov. from La Retama. A, MNCN-74495, left maxillar with DP2-M1 in buccal view; B, MNCN-74495, left maxillar with DP2-M1 in occlusal view; C, MNCN-74452, right M3 in lingual view; D, MNCN-74452, right M3 in buccal view; E, MNCN-74452, right M3 in occlusal view; F, MNCN-74450, left P4-M1 in lingual view; G, MNCN-74450, left P4-M1 in buccal view; H, MNCN-74450, left P4-M1 in occlusal view; I, MNCN-74451, right P3 in lingual view; J, MNCN-74451, right P3 in buccal view; K, MNCN-74451, right P3 in occlusal view; L, MNCN-74453, right P2 in buccal view; M, MNCN-74453, right P2 in lingual view; N, MNCN-74453, right P2 in occlusal view.

The DP2 and DP3 (Fig 3A and 3B) are triangular-shaped teeth with a well-marked rounded anterior lobe and a large lingual cone. Contrary to the DP2, there is a lingual cingulum around the base of the lingual cone in DP3. The buccal structures in both dental pieces are enormous and markedly triangular in shape. The DP4 is a molarized tooth that differs from the molars in being lower crowned and with more pyramid-like cusps with less developed cristae. The mesostyle and the parastyle are comparatively more developed than in molars. The anterior cingulum is moderately developed and the lingual cingulum is very weak.

Both the P2 and the P3 (Fig 3F–3N) have a similar morphology, being the P2 smaller and with a less protruding lingual cone. The P4 has a very robust buccal cone.

The molars (Fig 3C–3H) have round-based brachyodont cusps with moderately developed cristae. The mesostyle is large and the entostyle is variably developed. The post-protocrista is short, almost non-existent, with small enamel folds that disappear with wearing. The buccal cusps are not aligned but imbricated. The buccal rib of the metacone is huge. The post-metacrista is buccally folded in the M3, as occurs in *Triceromeryx pachecoi* (in which this feature is very exaggerated) and *Tauromeryx*. However, this condition is less marked in *Ampelomeryx*. The parastyle and the mesostyle are less developed than the buccal rib of the paracone. The upper molars have a metaconule-fold, but contrary to other species in which this fold is well marked forming a Y-shaped morphology (e.g. *T*. *pachecoi*), the anterior accessory fold of the metacone is very poorly developed or non-existent in *X*. *amidalae*. There are moderately developed anterior and lingual cingula.

#### Lower dentition

**Fig 4:**
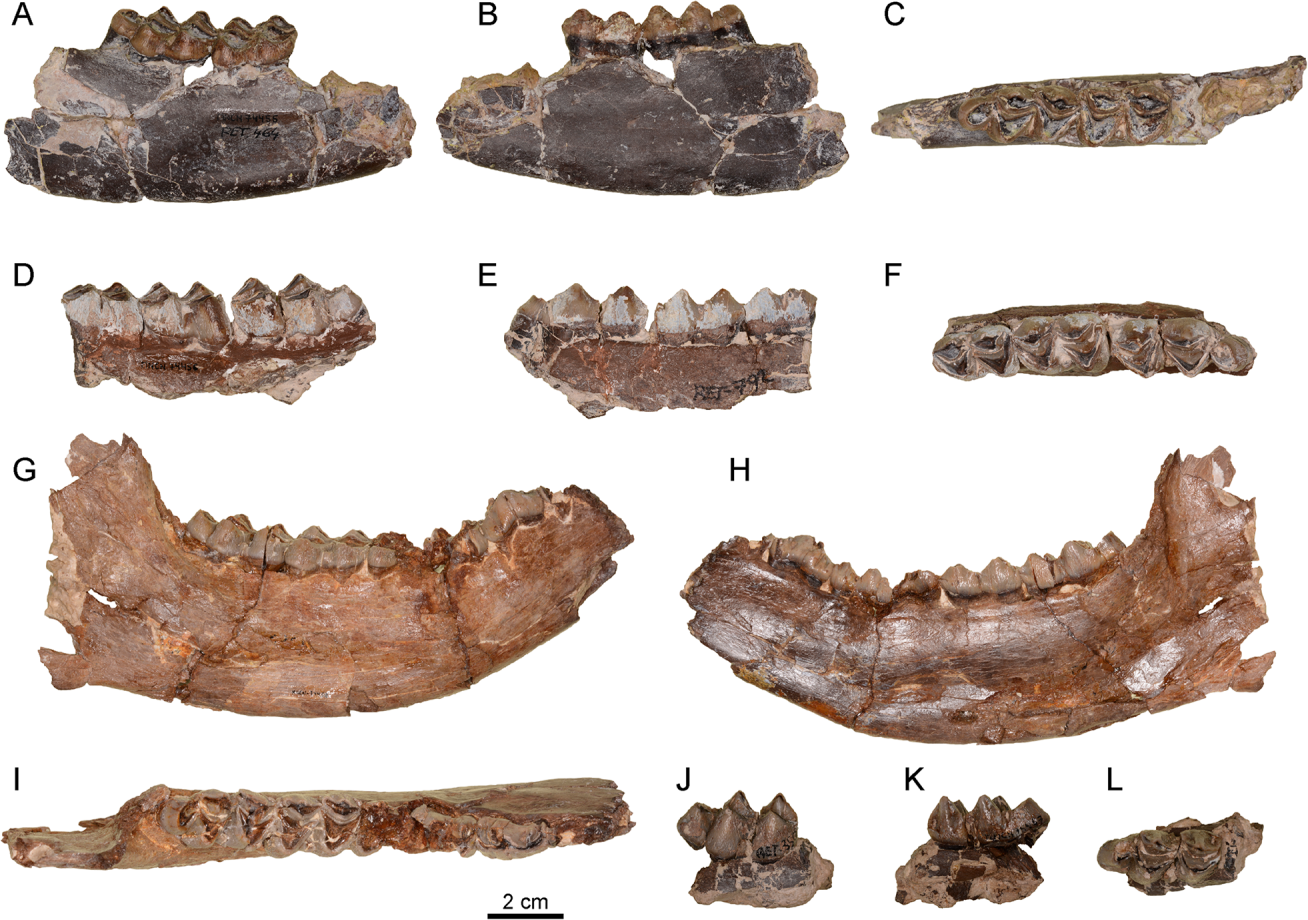
Lower dentition remains of *Xenokeryx amidalae* gen. et sp. nov. from La Retama. A, MNCN-74455, right hemimandibular fragment with m2-m3 in buccal view; B, MNCN-74455, right hemimandibular fragment with m2-m3 in lingual view; C, MNCN-74455, right hemimandibular fragment with m2-m3 in occlusal view; D, MNCN-74456, left hemimandibular fragment with m1-m3 in buccal view; E, MNCN-74456, left hemimandibular fragment with m1-m3 in lingual view; F, MNCN-74456, left hemimandibular fragment with m1-m3 in occlusal view; G, MNCN-74458, right hemimandible with p3-m3 in buccal view; H, MNCN-74458, right hemimandible with p3-m3 in lingual view; I, MNCN-74458, right hemimandible with p3-m3 in occlusal view; J, MNCN-74457, right m3 in buccal view; K, MNCN-74457, right m3 in lingual view; L, MNCN-74457, right m3 in occlusal view.

*Xenokeryx amidalae* has buno-selenodont molars with broad cuspids (Fig 4). The lingual cuspids are not aligned but imbricated. The *Palaeomeryx*-fold is short but robust, and spreads out directly from the protoconid (instead from the post-protocristid), disappearing with wearing. The tip of the post-metacristid shows, together with the metastylid, a T-shaped bifurcation that is not present in *Ampelomeryx*, *Triceromeryx* and *Tauromeryx*. The ectostylid and the metastylid are well developed. The anterior cingulid is more or less weak. The bi-cuspidate third lobe of the m3 is centrally oriented and has a robust hypoconulid. The enamel of the lower molars is slightly wrinkled.

### Postcranial skeleton

#### Scapula

**Fig 5:**
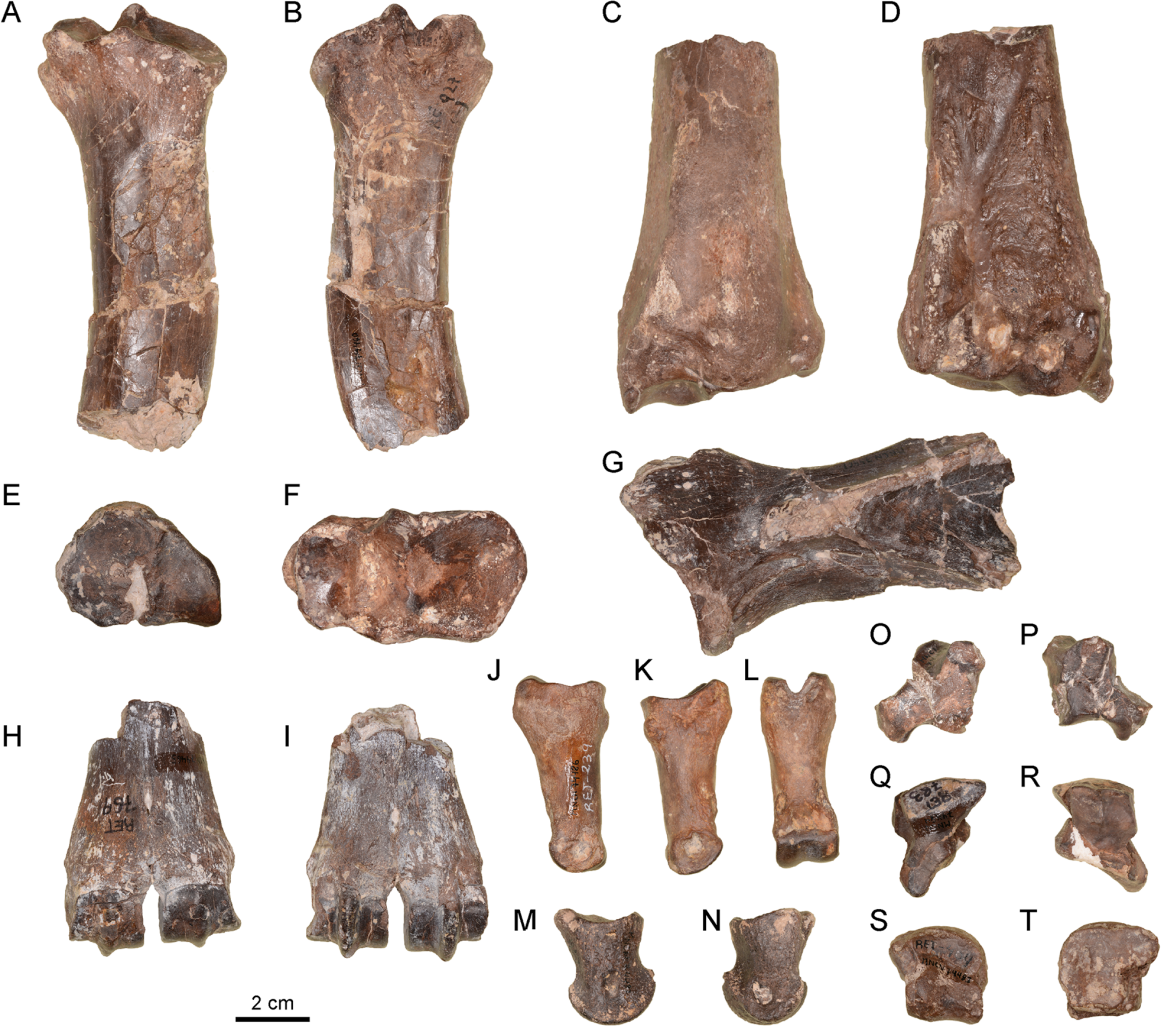
Fore limb remains and phalanges of *Xenokeryx amidalae* gen. et sp. nov. from La Retama. A, MNCN-74460, proximal fragment of right radius in cranial view; B, MNCN-74460, proximal fragment of right radius in caudal view; C, MNCN-74459, distal fragment of right radius in cranial view; D, MNCN-74459, distal fragment of right radius in caudal view; E, MNCN-74472, proximal fragment of metacarpal III-IV in proximal view; F, MNCN-74473, proximal fragment of left radius in proximal view; G, MNCN74461, articular fragment of left scapula in lateral view; H, MNCN-74465, distal fragment of metacarpal III-IV in dorsal view; I, MNCN-74465, distal fragment of metacarpal III-IV in palmar view; J, MNCN-74486, first phalanx in external view; K, MNCN-74486, first phalanx in interdigital view; L, MNCN-74486, first phalanx in palmar/plantar view; M, MNCN-74487 in external view; N, MNCN-74487 in interdigital view; O, MNCN-74493, right pyramidal in lateral view; P, MNCN-74493, right pyramidal in medial view; Q, MNCN-74484, right semilunate in proximal view; R, MNCN-74484, right semilunate in distal view; S, MNCN-74483, left magnotrapezoid in proximal view; T, MNCN-74483, left magnotrapezoid in distal view.

The only specimen (MNCN-74461; Fig 5G) is a distal fragment with almost only the articular area preserved. The glenoid cavity is elliptical. The supraglenoid tubercle is L-shaped. The acromion and the distal part of the scapular spine are not preserved. The distal end of the infraspinatous fossa is markedly triangular.

#### Radius / ulna

The trochlear-capitular facets for the humerus are cranio-caudally wide, giving the proximal articulation surface of the radius a rectangular shape (Fig 5A–5D and 5F). There is a triangular caudal notch between both facets, and the capitular facet lacks a caudal extension. The lateral ulnar facet contacts with the lateral trochlear gorge. The proximo-lateral insertion tubercle is cranio-caudally shorter than in *Triceromeryx*. In the distal articulation area the facet for the scaphoid shows a very pronounced convexity. The facet for the semilunar has a lateral notch, not so well-marked as in other pecorans sporting this feature, such as e.g. the moschid *Hispanomeryx* [26]. Contrary to *Triceromeryx*, the palmar region of the semilunar facet is short and ends into a deep groove. Interestingly enough, and also different from the condition in *Triceromeryx*, the distal portion of the ulna is fused to the radius in *Xenokeryx*.

#### Pyramidal

The proximal surface of the anterior process has a well-marked concavity. The facet for the pisiform is oval and concave, palmarly oriented. The distal process is long, occupying one third of the total length of the bone (Fig 5O and 5P).

#### Semilunate

The articular facet for the radius is T-shaped with a central constriction. The proximal facet for the scaphoid is subrectangular and concave. It does not contact with the elliptical dorsodistal facet for the scaphoid. The dorsal articular facet for the pyramidal is subcircular and flat. The distal facet for the pyramidal, located in the centro-lateral apophysis, is quadrangular and flat, almost dorsally oriented. The articular facets for the unciform and the magnotrapezoid occupy nearly identical portions of the distal articular surface (Fig 5Q and 5R).

#### Magnotrapezoid

*Xenokeryx* has a flat and wide magnotrapezoid (Fig 5S and 5T), very different from the tall, robust and narrow magnotrapezoid of *Triceromeryx* and *Tauromeryx*. There is a faint crest between the two proximal facets. The medial facet for the scaphoid is much larger than the facet for the semilunate. The former is quadrangular with a slight central constriction, whereas the latter is elongated and narrow, slightly broader on its dorsal end. The facet for the metacarpal III-IV is quadrangular in shape with a latero-dorsal extension different from the kidney-shaped facet present in *Triceromeryx*. This facet is flat and occupies almost all the distal surface of the bone. The dorsal facet for the unciform is narrow, small and dorso-palmarly elongated, different from the huge, short and triangular facet of *Triceromeryx* and other forms.

#### Metacarpal III-IV

The proximal articular surface is semicircular, with a large quadrangular facet for the magnotrapezoid (Fig 5E). The unciform facet is triangular in shape and smaller. There is a thin keel separating both facets that softens palmarly. The synovial fossa is well developed and elongated connecting palmarly to a groove that runs through the middle of the distal part of the diaphysis. There are two proximo-palmar rugose areas for the interosseous muscles. The preserved diaphysis (only a distal stretch) is slender (Fig 5H and 5I). The plantar surface above the distal articulation is convex. The tubercles for the collateral ligaments are well developed. The inter-trochlear incision is markedly V-shaped. There are no supra-articular fossetes.

#### Femur

**Fig 6:**
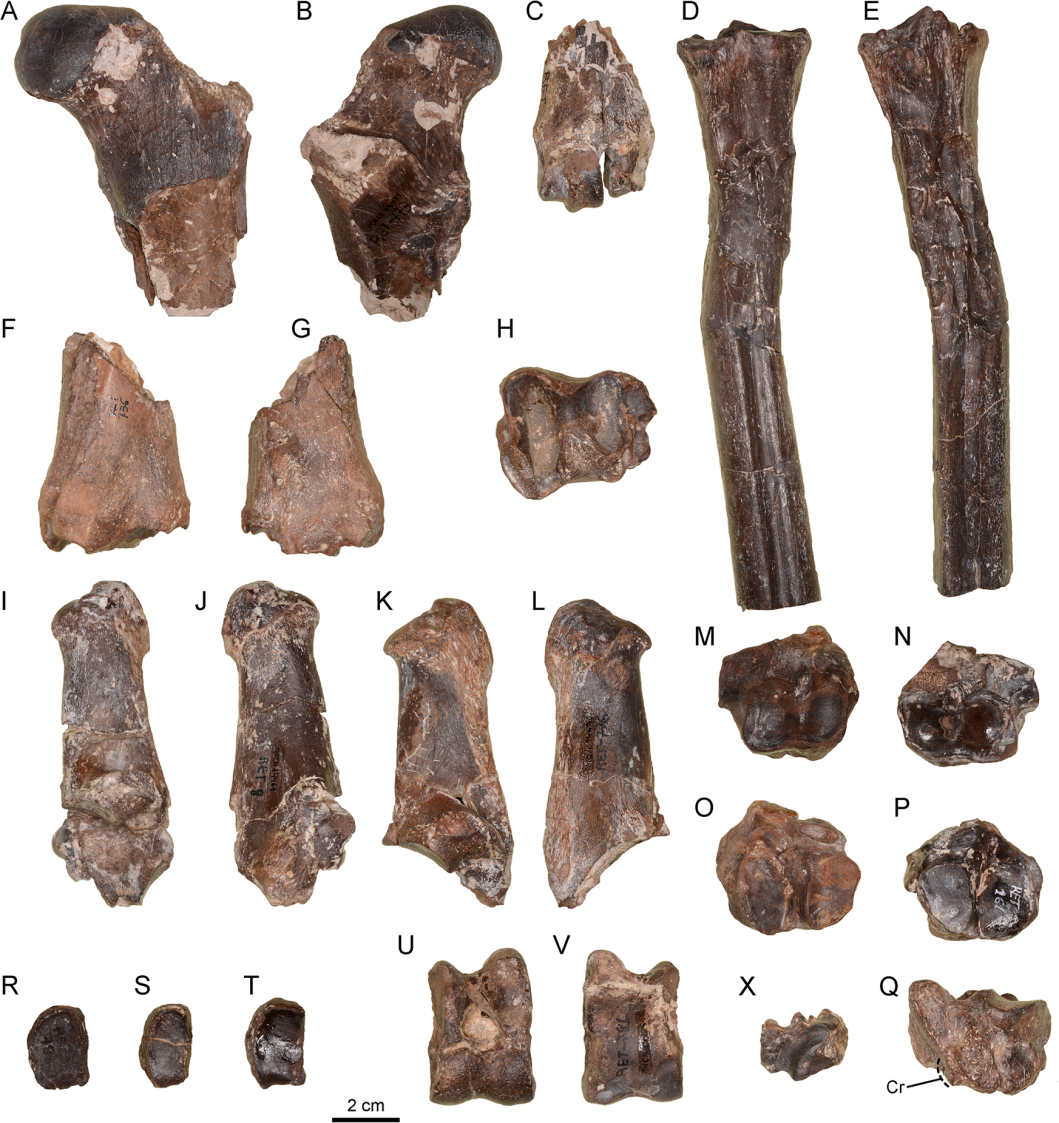
Hind limb remains of *Xenokeryx amidalae* gen. et sp. nov. from La Retama. A, MNCN-74471, proximal fragment of left femur in cranial view; B, MNCN-74471, proximal fragment of left femur in caudal view; C, MNCN-74475, distal fragment of metatarsal III-IV in dorsal view; D, MNCN-74464, proximal fragment and diaphysis of left metatarsal III-IV in dorsal view; E, MNCN-74464, proximal fragment and diaphysis of left metatarsal III-IV in plantar view; F, MNCN-74466, distal fragment of left tibia in cranial view; G, MNCN-74466, distal fragment of left tibia in caudal view; H, MNCN-74466, distal fragment of left tibia in distal view; I, MNCN-74469, right calcaneus in medial view; J, MNCN-74469, right calcaneus in lateral view; K, MNCN-74470, right calcaneus in medial view; L, MNCN-74470, right calcaneus in lateral view; M, MNCN-74477, right navicular-cuboid in proximal view; N, MNCN-74479, left navicular-cuboid in proximal view; O, MNCN-74477, right navicular-cuboid in distal view; P, MNCN-74479, left navicular-cuboid in distal view; Q, MNCN-74480, right navicular-cuboid in plantar view; R, MNCN-74502, right ectomesocuneiform in proximal view; S, MNCN-74501, right ectomesocuneiform in proximal view; MNCN-74500, right ectomesocuneiform in proximal view; U, MNCN-74476, left astragalus in dorsal view; V, MNCN-74476, left astragalus in plantar view; X, MNCN-74482, right malleolar in medial view. Abbreviations: Cr, crest.

The only specimen is a proximal fragment that includes the *caput femoris* and the femur neck (Fig 6A and 6B). The *caput femoris* is prominent and transversally elongated with a well-marked *fovea capitis*. The neck is narrow and well-marked. The trochanteric fossa is triangular and with developed borders. The small trochanter is robust and the inter-trochanteric line is very well marked.

#### Tibia

There are no proximal fragments in the sample (Fig 6F–6H). The fibular fissure is not very deep, but has well-marked borders. The lateral gorge of the tibial cochlea is slightly wider than the medial one. The malleolar facet is divided into a narrow cranial part and a rectangular wider caudal part that contact together under the distal end of the fibular fissure. A small crest separates the malleolar facet from the lateral gorge of the tibial cochlea. The surface of origin of the long medial collateral ligament, located just above the medial malleolus, is well marked. The *sulcus* for the digital medial flexor tendon is very well marked, deep and with developed cranial and caudal borders.

#### Malleolar

The middle proximal spine is short, wide and triangular, not surpassing the length of the dorsal and plantar spines (Fig 6X). The planto-distal articular surface for the calcaneum has a well-marked concavity. The medial facet for the astragalus presents a triangular flat central portion and a well-developed and smooth canal that has the shape of a quarter of circumference.

#### Calcaneum

In proximal view the *tuber calcis* is hexagonal, with a wide and rounded dorsal apex (Fig 6I–6L). The plantar crests for the insertion of the *gastrocnemius* tendon are wide and fuse into a distal triangular structure. The dorsal and plantar borders of the *corpus* are convergent. The *sustentaculum tali* is well developed with a strong medial projection. The malleolar facet has a prominent convex proximal part and a smaller and flatter distal part. The distal facet for the navicular-cuboid is elongated and slightly concave. The main facet for the astragalus is wide and has a slight central convexity.

#### Astragalus

The lateral condyle of the proximal trochlea is wider and higher than the medial condyle (Fig 6U and 6V). The plantar trochlea occupies almost the entire plantar surface of the astragalus and connects along the distal border with the articular surface of the distal trochlea. The lateral border of the distal trochlea is straight, lacking a notch. This is also the case in *Tauromeryx*, but not in *Triceromeryx*. Both trochleae are equally sized.

#### Navicular-cuboid

There are several well-preserved specimens (Fig 6M–6Q). The articular surfaces for the distal trochlea of the astragalus are similarly wide but the medial one is longer, extending over the proximo-plantodistal process. In the plantar side of this process there is a well-marked crest that does not reach the proximal half of the process, very similar to that present in giraffoids as climacoceratids and giraffids (although in giraffids is more developed). Lateral to this crest there is a canal, triangular and with marked borders, that is not so well developed as in giraffids. The dorsal facet for the metatarsal is kidney-shaped to elliptical, with a more or less developed internal notch depending on the specimen. The plantar metatarsal facet is slightly convex, elongated and small, separated from the dorsal metatarsal facet by a deep groove, and lying horizontally. The articular facet for the ectomesocuneiform is oval and slightly convex, and the facet for the entocuneiform is much smaller, rounded to elliptical and concave.

#### Ectomesocuneiform

The facet for the navicular-cuboid is concave and subrectangular to elliptical, extending slightly on the proximo-plantar area of the bone. The facet for the metatarsal III-IV is slightly convex and elliptical (Fig 6R–6T).

#### Metatarsal III-IV

The proximal surface is pentagonal in shape. The main facet for the navicular-cuboid is sub-triangular with a slight central convexity. The small plantar facet for the navicular-cuboid is small and narrow, elongated and inclined. The facet for the ectomesocuneiform is kidney-shaped and slightly concave. The facet for the encotuneiform is sub-triangular and much smaller than the facet for the ectomesocuneiform. The furrow for the lateral extensor tendon is relatively short (Fig 6D and 6E). The lateral metatarsals (II and V) are clearly fused proximally to the metatarsal III-IV. There is a short and rounded plantar metatarsal tuberosity, not as elongated as in moschids, cervids and dromomerycids. The metatarsal *sulcus* is distally closed, and the canal for the common artery is of ‘moschid-type’ [26]. The inter-trochlear incision is V-shaped. There are no plantar terminal fossetes over the distal articular keels (Fig 6C). There are no supra-articular fossetes.

#### First phalanx

The only first phalanx in the sample is well preserved (Fig 5J–5L). The central *sulcus* of the proximal articular surface is deep and does not open dorsally. The external facet is subtriangular and wider than the rectangular internal facet. The furrow for the tendon of the *interosseus* muscle is extremely faint. However there is a robust bulge for ligamentous attachment (carpo-metacarpal or tarso-metatarsal) in the dorso-internal part of the phalanx, just under the proximal articular surface. *Triceromeryx* and *Tauromeryx* present this same bulge (albeit smaller) in the dorso-external part of the phalanx instead. The insertion area for the interdigital ligament is elliptical and faint. The plantar / palmar border is straight, with the plantar concavity located just under the proximal epiphysis. In dorsal view the external face of the phalanx is concave. The distal articulation facet does not extend very deeply into the flexor area, having a straight flexor border, with no expansions.

#### Second phalanx

This phalanx is short and robust (Fig 5M and 5N). The external proximal articular facet is slightly larger than the internal one. The post-articular plateau is developed. The extensor process is short and blunt. The insertion area for the *flexor digitorum superficialis* tendon is triangular and marked. In interdigital view the distal articular facet is angled instead of rounded.

## Results of the Phylogenetic Analyses

### First analysis: *Xenokeryx* among palaeomerycids

**Fig 7:**
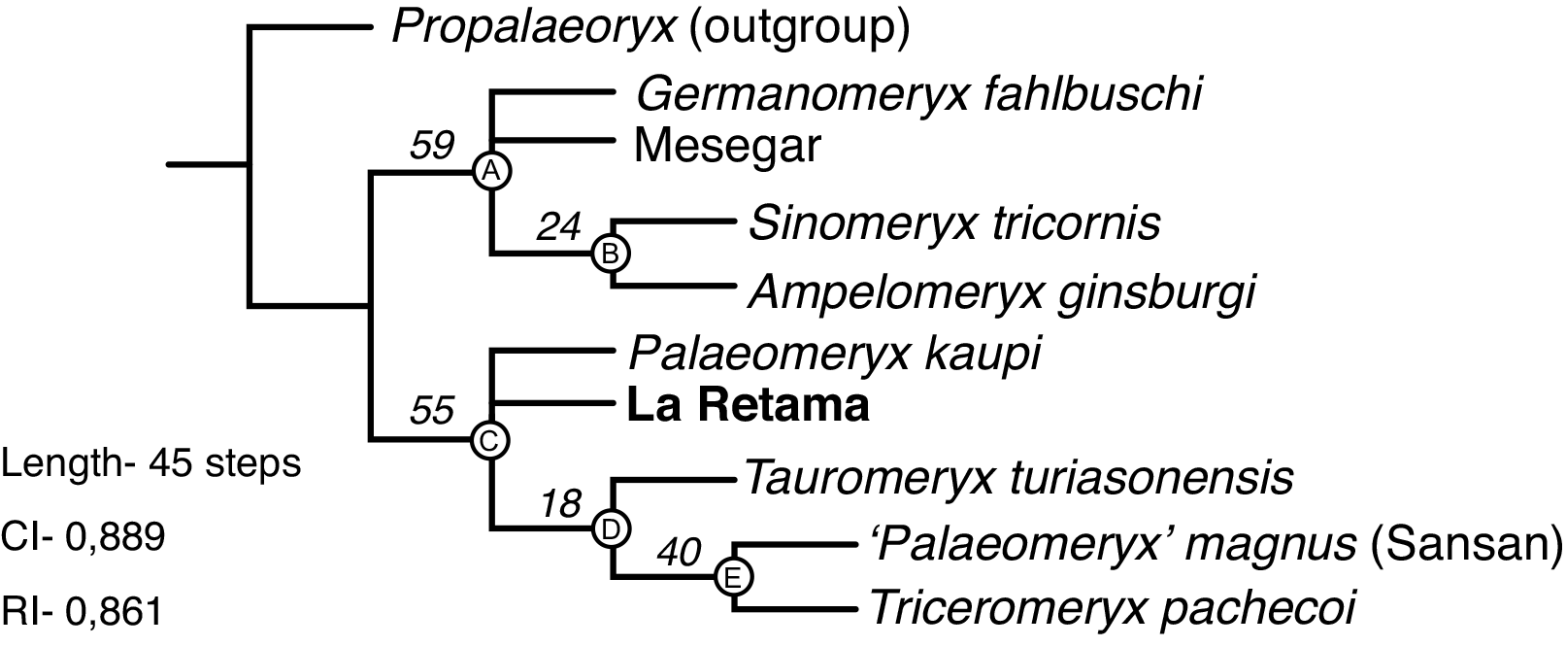
Phylogenetic relationships among palaeomerycids. Bootstrapped MPT of 45 steps (CI = 0,889; RI = 0,861). Bootstrap values (1000 replicates) over each node.

The Maximum Parsimony search produced a single most parsimonious tree (MPT) of 45 steps (CI = 0.889; RI = 0.861; Fig 7) with a basal dichotomy that divided the included palaeomerycids into two well-differentiated clades, one of them including *Ampelomeryx*, *Sinomeryx* and *Germanomeryx*, and the other one including *Triceromeryx*, *Palaeomeryx*, *Tauromeryx* and *Xenokeryx*. The distribution of character states for the internal nodes and the autapomorphies of the terminals are presented in Table 1. Within the first clade there is a basal politomy formed by *Germanomeryx*, Mesegar-2 and a clade composed by *A*. *ginsburgi* and *S*. *tricornis*. In the second clade a basal politomy exists between *P*. *kaupi*, *X*. *amidalae* and a clade containing *T*. *turiasonensis* as a basal offshoot and ‘*P*.’ *magnus* + *T*. *pachecoi* as sister terminals. Some of the branch supports are weak (more fossil data are needed to fill the missing data, especially in the *Ampelomeryx* group).

**Table 1:** *Xenokeryx* and palaeomerycids, distribution of autapomorphic character states for the internal nodes and *Xenokeryx*. Ambiguous synapomorphies in italics.

Node / Taxon	Character (State)
Node A	*8(1)*; 9(3); 15(0); 22(1); 24(1)
Node B	1(0); 2(0); 3(0); 6(0); 14(0); 16(1); 17(0)
Node C	*1(1)*; *2(1)*; 3(1); 6(1); 8(0); *14(1)*; 18(1); 19(1); 20(1); 21(1); 24(0)
Node D	17(0); 26(0); 32(1)
Node E	27(1); 30(0)
*Xenokeryx amidalae*	9(2); 12(2); 13(2); 28(1); 29(0); 30(1); 31(1); 32(2)

### Second analysis: Palaeomerycids within the Pecora

**Fig 8:**
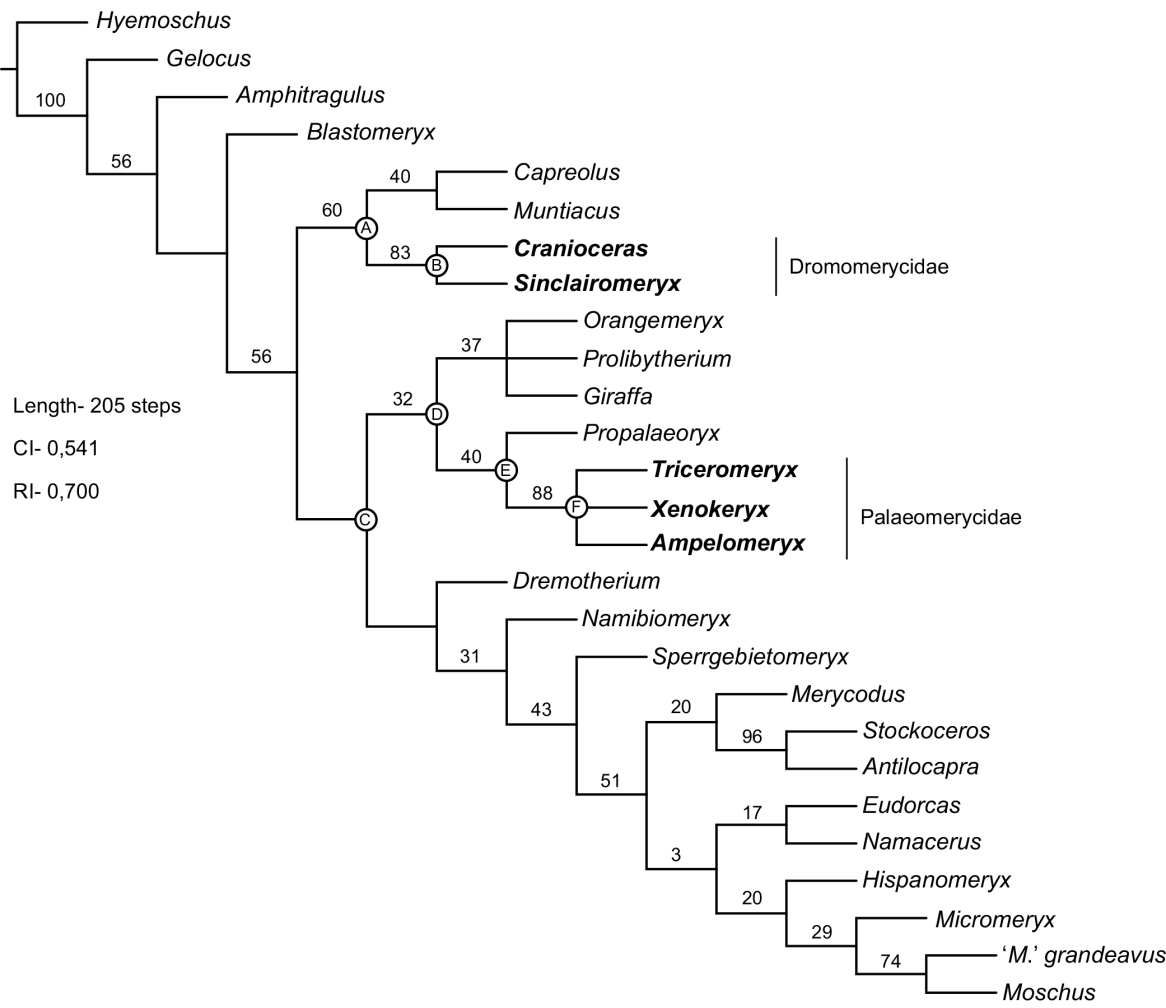
Phylogenetic relationships of palaeomerycids within the Pecora. Bootstrapped MPT of 205 steps (CI = 0,541; RI = 0,700). Bootstrap values (1000 replicates) over each node. Dromomerycid and palaeomerycid terminals are highlighted.

**Fig 9:**
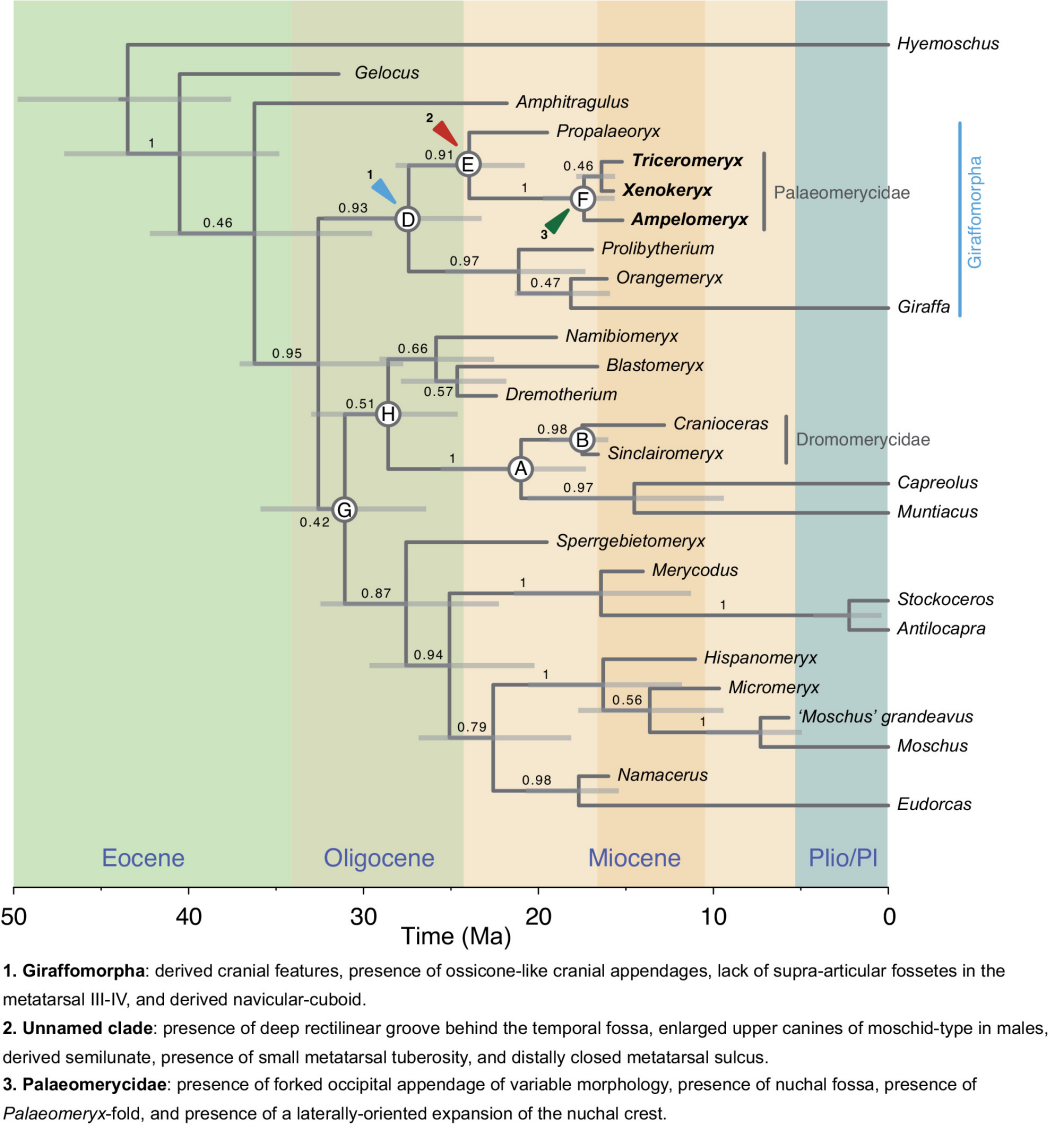
Phylogenetic relationships of palaeomerycids within the Pecora. Maximum clade credibility time-tree of major ruminant clades obtained from our ‘tip-dating’ Bayesian analysis. Uncertainty in divergence ages is shown with horizontal grey bars representing their 95% highest posterior densities (HPDs). Numbers above each branch show the posterior probability (PP) of the respective clade.

Both the MP and Bayesian analyses recovered similar topologies (Figs 8 and 9), remaining fully consistent among them in the relationship of palaeomerycids with their immediate sister groups, and also in assessing their position with respect to dromomerycids. The Maximum Parsimony search produced a single most parsimonious tree (MPT) of 205 steps (CI = 0.541; RI = 0.700). However the branch support is weak in general. Both the distribution of character states for the selected internal nodes of the MPT and the reconstructed states at the selected internal nodes of the Bayesian tree are presented in Tables 2 and 3. In all solutions (MP-morphological and Bayesian-combined) *Gelocus* and *Amphitragulus* are always placed basal to a clade of derived pecorans that diverged in the Oligocene (around 32 Ma) into three main clades that are recovered from both the MP and Bayesian analyses (with posterior probabilities, PP, above 0.5): a giraffomorph-clade, a cervoid-clade and a bovidomorph-clade including bovoids (moschids + bovids) and the antilocaprid-like forms (merycodonts + antilocaprids) plus their stem groups. In all cases the Palaeomerycidae clusters with *Propalaeoryx* as the closest sister group and with a Giraffoidea composed by *Prolibytherium* plus a clade comprising *Giraffa* and *Orangemeryx*, whereas the Dromomerycidae groups with the Cervidae. The Bayesian analysis shows high posterior probability for both the dromomerycid + cervid (PP = 0.98) and the palaeomerycid-*Propalaeoryx* + giraffoid (PP = 0.93) clades (named as the Giraffomorpha herein). According to our tip-dating Bayesian reconstruction, giraffomorphs, bovidomorphs and cervoids would have originated at the end of the Oligocene (ca. 29–27 Ma). Despite the uncertainty of the divergence times yielded by our analysis, the confidence intervals of these three basal nodes mainly fall within the Oligocene (between 33–24 Ma). Only the confidence interval on the basal bovidomorph node would be compatible with a very early Miocene origin.

**Table 2:** Palaeomerycids within the Pecora, distribution of character states for the discussed internal nodes of the MPT. Ambiguous synapomorphies in italics.

Node / Taxon	Character (State)
Node A	6(1); 19(0); 53(2); 56(1)
Node B	13(6); 23(1); *35(1)*; 41(1); 43(2)
Node C	17(1); 20(1); 54(0); 64(0)
Node D	9(4); 10(1); 12(1); 13(2); 35(3); 57(0); 62(2)
Node E	25(1); *39(2)*; 56(2)
Node F	18(1); 24(1); *46(0)*; 67(1)

**Table 3:** Palaeomerycids within the Pecora, distribution of the reconstructed morphological character states for the discussed internal nodes of the Bayesian tree. Ambiguous synapomorphies in italics.

Node / Taxon	Character (State)
Node A	6(1); 15(1); 17(2); 20(0); 53(2); 56(1); 64(1)
Node B	13(6); 23(1); *35(1)*; 41(1); 43(2); *51(1)*; *61(0)*
Node D	9(4); 10(1); 12(1); 13(2); *20(1)*; *54(0)*; *57(0)*; 62(2)
Node E	25(1); *39(2)*; 51(1); 56(2); *58(1)*
Node F	18(1); 24(1); 46(0); 67(1)
Node G	14(1)
Node H	19(0); *58(1)*

There are several differences between the MPT and the Bayesian summary tree. The first one is the relative position of the giraffomorph clade with respect to the other pecorans. Whereas giraffomorphs cluster with bovidomorphs in the MP analysis, they interchange its position with the cervoids (cervids + dromomerycids) in the combined Bayesian analysis splitting as the basal offshoot of the large clade of derived pecorans (although this cervoid + bovidomorph clade was recorded with a PP = 0.42). Also, a relatively well-supported (and very interesting) sister group to cervoids composed by *Namibiomeryx* and *Blastomeryx* plus *Dremotherium* is recovered in the Bayesian analysis (PP = 0.66). These stem cervoids appear however as a part of the bovidomorph clade in the MPT. Taken together, our phylogenetic results support both the hypothesis of palaeomerycids and dromomerycids not being sister groups and that of palaeomerycids and giraffids being closely related, thus excluding the former from the cervoid lineage. Finally, apart from the overall good branch support of the Bayesian combined tree, the synapomorphic states reconstructed for both the clade *Propalaeoryx* + Palaeomerycidae and the more inclusive giraffomorph clade are more numerous than their counterparts in the MPT, so as pointed by Lee [56] the Bayesian analysis appears to be less sensitive to homoplasy than traditional parsimony. The inclusion of DNA data also adds robustness to the Bayesian results. Thus the discussion of our results is based on the Bayesian topology unless noted otherwise. As a final comment, these analyses are not intended to produce a full phylogenetic hypothesis of the Pecora but the overall results are a good frame for future research.

## Discussion

### Phylogenetic position of palaeomerycids within the Pecora

The Palaeomerycidae is a monophyletic group of pecorans diagnosed by the presence of a single forked occipital appendage formed by the elongation of the supraoccipital and the nuchal plane, presence of nuchal fossa, *Palaeomeryx*-fold in the lower molars, and a laterally-oriented expansion of the nuchal crest. As we will discuss later, our phylogenetic hypothesis reconstructs the presence of ossicones as a basal feature of all the giraffomorphs. We define the Palaeomerycidae as the least inclusive clade of pecorans containing *Triceromeryx* and *Ampelomeryx*. Much has been written on the *Palaeomeryx*-fold, also known by the more ‘neutral’ name of external post-protocristid [71], as a primitive and unique structure that disappears in the more advanced forms within the different pecoran lineages. Our present work helps to reject this pre-conception and show the *Palaeomeryx*-fold (or better said the different *Palaeomeryx*-folds, because several morphologies of this post-protocristid fold exist) as a structure that has appeared several times within different unrelated lineages of pecorans (e.g. palaeomerycids and moschids) and has also been secondarily lost in others (e.g. cervids). The *Palaeomeryx*-fold is not the only known dental structure of pecorans that secondarily appears in a given lineage. The case of the metastylids and other dental structures of the moschid *Hispanomeryx andrewsi* [45] perfectly pictures how plastic the pecoran dentition can be, and how supposedly ‘primitive’ and previously lost dental structures can be ‘regained’ into a clade of relatively derived pecorans. Palaeomerycids are also characterized by a highly modified occipital area. The nuchal fossa and the expansion of the nuchal crest were never described before and are related with an extension of the surface area for the insertion of the neck musculature, both longitudinally (nuchal fossa) and laterally (expansion of the nuchal crest). The latter allows for a more pronounced lateral bounding of the head. As commented in the description of *Xenokeryx*, the nuchal fossa receives the insertion of the extensors muscles *rectus capitis dorsalis*, *semispinalis capitis* and the *rectus capitis dorsalis minor*. As such, the longitudinal expansion of all these muscle packs would allow for a more powerful head extension. Also, as noted by Astibia et al. [24] the neck musculature probably climbed the most basal part of the occipital appendage acquiring a relatively pronounced angle in its occipital insertion, thus helping in the enhanced head extension of palaeomerycids. These neck-head modifications resulted in powerful lateral and dorsal movements of the head although their exact purpose is not known, and both ecological and behavioral morpho-functional hypotheses could be suggested (e.g. male intraspecific fighting is an obvious one) but we have no data to back up any of them.

We support the hypothesis of a very close relationship between palaeomerycids and giraffoid pecorans (the clade that includes *Giraffa* and *Prolibytherium*, their more recent common ancestor and all of its descendants). The Giraffomorpha is defined here as the least inclusive clade containing *Giraffa* and *Triceromeryx*. We reject the assignment of *Prolibytherium* to the Palaeomerycidae proposed by several authors [10,27,29], and confirm its arrangement within the Giraffoidea [11, 26]. However, *Prolibytherium* does not cluster with the Climacoceratidae, so we also reject our previously proposed hypothesis of a sister-group relationship between *Prolibytherium* and the true climacoceratids such as *Orangemeryx* [26].

As we already commented, the systematics and phylogenetic relationships of palaeomerycids were controversial. Many authors [4,5,13,27,29,34,35,72] considered the Palaeomerycidae as members of the Cervoidea (the pecoran forms more related to cervids than to another of the extant ruminant families). Janis & Scott [27], which offered an extensive revision of the Cervoidea, supported the cervoid affinities of palaeomerycids on the presence of distally closed metatarsal sulcus (a character widely used by other authors as well), presence of *Palaeomeryx*-fold in the lower molars, sabre-like upper canine in males, and presence of plantar metatarsal tuberosity in the metatarsal III-IV. However, palaeomerycids and giraffoids form a well-supported clade of giraffomorph pecorans in our phylogenetic tree (PP = 0.93). The hypothesis of relationship of palaeomerycids and giraffoids was already proposed by several authors mainly on the basis of the presence in both groups of ossicones and a suite of cranial, dental and postcranial characters [15,21,36]. However Janis & Scott [27] dismissed this hypothesis arguing that all these characters (including the presence of ossicones) were convergences, but Solounias [10] again resurrected the presence of ossicones as a feature that probably related palaeomerycids and giraffoids. In the meantime, Ginsburg [28] related palaeomerycids with dromomerycids and this group with giraffids and bovids, including all of them in the Bovoidea. That work was a good example of how almost all possible hypotheses of relationship were suggested for palaeomerycids. The reconstructed synapomorphies that link palaeomerycids with giraffoids in our tree include both cranial (morphology of the retroarticular process; contact between the retroarticular process and the external acoustic tube; laterally enclosed temporal canal; well-marked lateral margin of the infratemporal fossa; and presence of ossicones) and postcranial features (central plantar column of the metatarsal III-IV; absence of supraarticular fossetes in the metatarsal III-IV; and presence of a well-developed crest in the planto-medial area of the navicular-cuboid that does not reach the proximal region of the planto-medial process). The distribution of the condition of the metatarsal *sulcus* among pecorans cannot be used alone to link cervids with other groups. Palaeomerycids display a ‘moschid-type’ disposition of the *sulcus* for the common digital artery [26], which is the most common state among pecorans. When the digital artery is of moschid-type (superficial but not as superficial as in crown bovids), both conditions of the *sulcus*, open and closed, are expected to appear (e.g. the case of the Moschidae is archetypical of this). The true cervoid condition (see character 53) is the presence of a deep *sulcus* (cervid-type) that runs through the very middle of the shaft and fixes the distally closed condition of the gully. Hence, as occurs with moschids [26] the distal closing of the gully (character 58) is a parallelism between palaeomerycids and the inclusive clade that contains cervoids and their stem hornless forms (node E; Fig 9). Also, as in [26] the presence of a metatarsal tuberosity (character 56) does not appear in this work as a unique condition of cervoids since it is also present as a parallelism in other non-cervoid groups (e.g. moschids). In addition, in palaeomerycids and their closest sister-group *Propalaeoryx* this proximo-plantar tuberosity in the metatarsal III-IV is not as developed as in cervids, dromomerycids and moschids. Regarding the enlarged sabre-like canines of males, palaeomerycids had a moschid-type canine (with a characteristic double curvature;[26]), being this morphology basal and widespread among pecorans. Moreover, cervids (with the exception of the secondarily ‘fanged’ *Hydropotes*) possess a derived kind of enlarged canines that lack the double curvature of the moschid-type (cervid-type). The morphology of the plantar surface of the navicular-cuboid appears to be of the upmost importance for grouping the giraffomorphs together in our phylogenetic tree. This is also the case with the bovidomorphs, that show a characteristic featureless plantar surface of the navicular-cuboid, but this group is not to be discussed here. Giraffomorphs possess a well-developed crest that rise from the distal part of the planto-medial surface of the navicular-cuboid but does not reach the proximal border. This feature (character 62) becomes very exaggerated in giraffids, the navicular-cuboid of which has a complete crest. Also, the canal that runs laterally to this crest is more developed, with a marked concavity, in the Giraffoidea. Giraffomorphs are also characterized by the absence of supra-articular fossetes in the metatarsal III-IV (character 57). Analogous structures to the supra-articular fossetes of pecoran ruminants have been described in other ‘ungulate’ groups and related to improved running abilities [42] due to the augmented extension capability of the phalanges that enhance the elastic charge of the *interosseus* and flexor tendons that run on the plantar side of the feet. However this is difficult to quantify and we do not know the influence of the presence / absence of supra-articular fossetes in the biomechanics and running capabilities of pecorans. Nevertheless, it seems that the absence of these structures is a fairly good phylogenetic signal for giraffomorphs. Interestingly enough, the presence of ossicones (character 13) is recovered as a basal feature for the Giraffomorpha and not as a parallel development in giraffids and palaeomerycids. This phylogenetic reconstruction establishes an evolutionary hypothesis that implies a basal homology of the appendages of *Prolibytherium* and climacoceratids with the ossicones despite their disparate external morphologies, both coded here as different character states [2,12]. This is a very interesting question that could only be fully answered through a comparative histologic analysis of the supra-orbital cranial appendages present in every giraffomorph group from which these cranial structures are known.

The Eurasian palaeomerycids share a common ancestor with the Miocene African pecoran *Propalaeoryx*. Remains of this genus have been found in both South and East Africa [11, 30, 48, 72]. It was accepted that *Propalaeoryx* was a member of the Giraffoidea [11,27,30,48] with some authors regarding it as a climacoceratid within giraffoids [11,48], an exclusively African family that contains forms such as *Orangemeryx*, included in this work. *Propalaeoryx* appears with a high support as the closest sister-group to the Palaeomerycidae (PP = 0.91), and hence we reject the hypothesis of *Propalaeoryx* belonging to both the Climacoceratidae and the Giraffoidea. Apart from postcranial features such as the distally closed metatarsal gully and the previously commented small version of the metatarsal tuberosity, the most intriguing of the derived traits shared by palaeomerycids and *Propalaeoryx* is the presence of a deep dorso-ventral rectilinear groove located between the caudal part of the temporal fossa and the nuchal plane (character 25). The function of this groove is unknown, although it strongly resembles a robust superficial vascular canal. The close relationship of palaeomerycids and *Propalaeoryx* probably implies a vicariance event that took part in the Oligocene / Miocene boundary (~24 Ma) that split-off the original common lineage into two branches, African and Eurasian. Thus, the evolutionary history of giraffoids-giraffomorphs (and of palaeomerycids themselves) results more complicated than previously thought. Whereas palaeomerycids preserved a more primitive type of dentition, *Propalaeoryx* shows a mosaic pattern of derived dental traits (more flattish and higher-crowned cuspids) with primitive features as the retention of the p1 [11,30]. As of today, there is no evidence of cranial appendages in *Propalaeoryx*, and resolving this issue with future discoveries should clarify the pattern of evolution of the supra-orbital appendages of giraffomorphs.

A clade containing dromomerycids and cervids placed within a more inclusive clade well differentiated from giraffomorphs is well supported in our phylogenetic tree (P = 1.0), thus corroborating the hypothesis of dromomerycids and palaeomerycids not being sister groups or even closely related into a major inclusive clade. We reject the proposals of Janis & Scott [27] and Prothero & Liter [29] that regarded the frontal appendages of palaeomerycids and dromomerycids as ‘labile and variable’ within a ‘family Palaeomerycidae’ that contained both ‘palaeomerycines’ and ‘dromomerycines’. Dromomerycids have supra-orbital appendages that never show the macroscopic features and suture with the skull roof associated with giraffid and palaeomerycid ossicones. They probably represent apophyseal structures [1], but a histological study of these supra-orbital appendages is needed to comprehend their true nature. The occipital appendage of dromomerycids is also completely different in morphology and probably in origin. First of all, it is not universally present in the Dromomerycidae, whereas all known cranial remains of male palaeomerycids with preserved occipital area present the occipital appendage. In dromomerycids this element is a sub-cylindrical structure that grows up from the supra-occipital (at the end of the sagittal line of the cranium) creating a single, non-forked rod. In palaeomerycids the occipital appendage not only involves a more or less vertical growth of the supra-occipital area, but also a lateral expansion that results in the integration of areas belonging to the occipital crest and in the development of an appendage that is not the cylindrical rod of dromomerycids but a laterally expanded structure that becomes broad and elliptical or flattish in cross-section [24]. Also, the nuchal plane gets reorganized, extending upwards and forming the well-developed pit that we name here as the nuchal fossa. However, in dromomerycids this kind of reorganization does not occur and the nuchal plane presents the rectilinear and concave morphology (with a subtle crest in the sagittal plane) typical of ruminants. Apart from the cranial appendages, dromomerycids are set apart from palaeomerycids by a huge set of cranial, dental and postcranial derived features (Nodes E, F and G; Table 3, Fig 9).

### Phylogeny and evolutionary history of palaeomerycids

**Fig 10:**
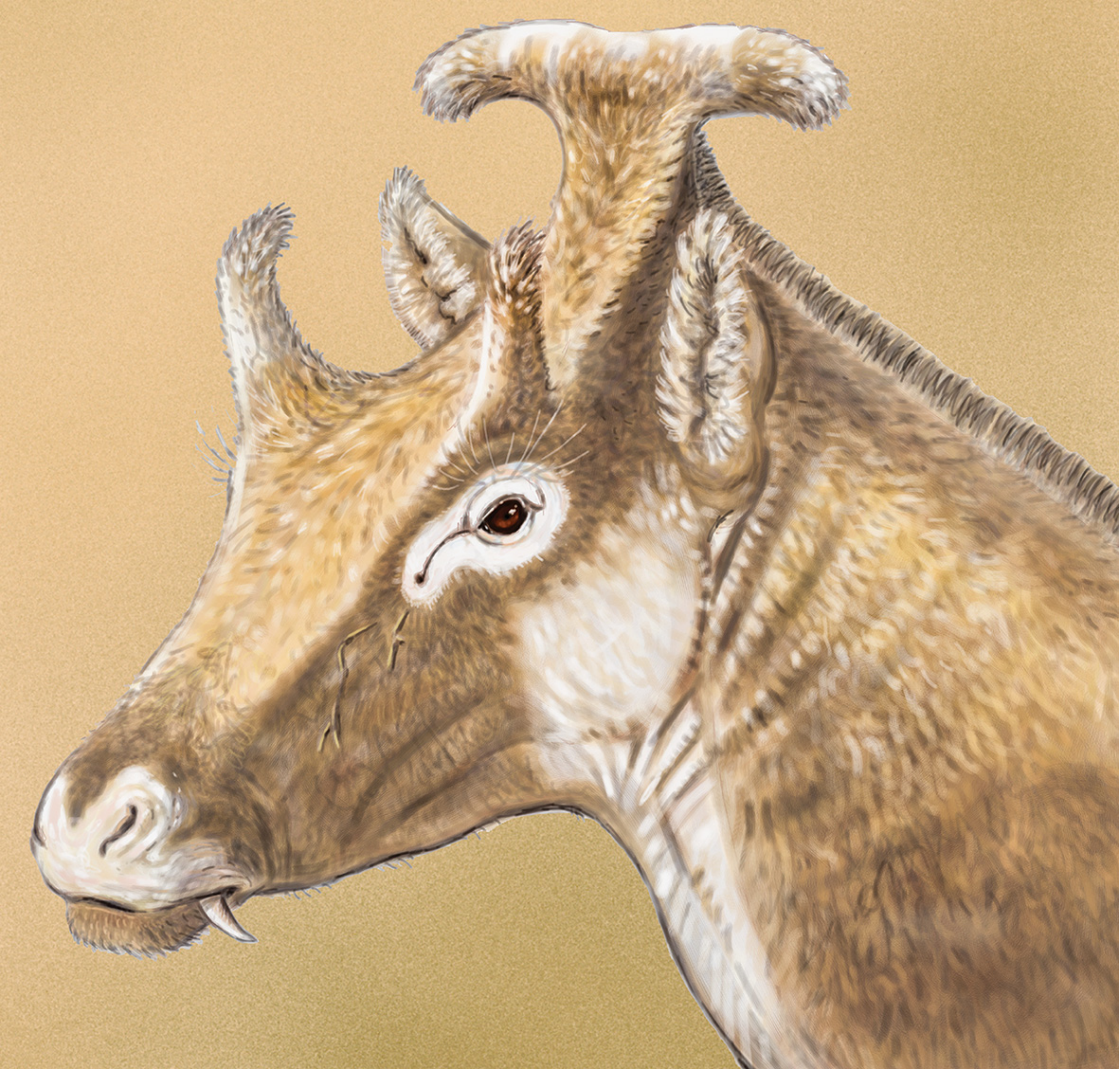
Life reconstruction of the head of *Xenokeryx amidalae* gen. et sp. nov. Adult male based on the fossils from La Retama. Illustration by IMS.

There are two main lineages of palaeomerycids. Clade A (*Ampelomeryx*-clade; Figs 8 and 10) includes ‘*Germanomeryx’ fahlbuschi*, ‘*Sinomeryx’ tricornis*, Mesegar-2 and *Ampelomeryx gingsburgi*, their last common ancestor and all of its descendants. On the other hand, the *Triceromeryx*-clade (clade C) is defined as *Palaeomeryx kaupi*, *Xenokeryx amidalae* (Fig 10), *Tauromeryx turiasonensis*, ‘*Palaeomeryx’ magnus* and *Triceromeryx pachecoi*, their last common ancestor and all of its descendants. *Ampelomeryx*-clade palaeomerycids are diagnosed by having an Y-shaped and narrow occipital appendage of variable length, elongated and large nuchal extension, well developed *Palaeomeryx*-fold, elongated and buccally positioned hypoconulid in the m3, and winged labial cone in the P4. Clade B contains ‘*Sinomeryx’ tricornis* + *Ampelomeryx ginsburgi*, and is characterized by flattish, non-pneumatized ossicones with an anteriorly positioned extension ‘wing’, presence of frontal eyebrow-like projections at the ossicone base, nuchal crest extended into the shaft of the occipital appendage, and a simple distal end of the post-metacristid. However, the ossicones of the two basal taxa ‘*G*.’ *fahlbuschi* and Mesegar-2 are unknown, so it could be very plausible that all or some of the ossicone features of clade B (characters 1, 3 and 6) would be in fact characterizing the entire *Ampelomeryx*-clade. Clade C (*Triceromeryx* clade) is characterized by a good number of cranial and dental features, such as the presence of cylindrical and pneumatized ossicones, extension ‘wing’ posterior to the ossicone (secondarily lost in *Tauromeryx*), short and triangular nuchal expansion, broad occipital appendages, p4 larger and more triangular than the p3, short or almost absent *Palaeomeryx*-fold and a suite of p4 derived traits.

A more derived lower dentition and a more diverse array of occipital appendage shapes and sizes are features that distinguish the members of the *Triceromeryx*-clade from those of the *Ampelomeryx*-clade. The occipital appendages in the *Ampelomeryx*-clade are always variations of a flat, more or less elongated, broadly pointed and almost horizontal structure, whereas the occipital appendages in the *Triceromeryx*-clade are autapomorphic for each genus, having their own characteristic features [21, 22, 24]. Both *Xenokeryx* and *Triceromeryx* have upright structures with an elliptical and broad pedicle, but the overall morphology is totally different: T-shaped with downwards-oriented branch tips and flat posterior surface in *Xenokeryx* versus Y-shaped appendages with enormous rod-like posterior crests in *Triceromeryx*. Moreover, *Tauromeryx* has its own type of occipital appendages that consist in very short and flattish Y-shaped structures with small points. Also, the lower dentition is essential to discriminate between the two clades. The *Ampelomeryx*-clade has a more primitive dentition with well-developed *Palaeomeryx*-fold, whereas the *Triceromeryx*-clade accumulates several derived traits in the premolars and has a reduced *Palaeomeryx*-fold. These characters can be easily used to identify members of one clade or the other. However the dentition cannot be used to distinguish between taxa because all members of the *Ampelomeryx*-clade have the same type of dentition and the same occurs with all members of the *Triceromeryx*-clade. This is the reason behind the status of *species inquirenda* for the fossil remains from Georgensmünd, in absence of cranial appendages and due to the variability of the occipital structures within this clade. Contrary to the *Triceromeryx*-clade, that incorporates the morphology of the occipital appendages as autapomorphic features of the terminals, the *Ampelomeryx*-clade, as previously commented, concentrates the general morphology of the occipital appendages (and also probably that of the frontal ones, although this must be checked through future discoveries) in the base of the clade, with ‘minor’ differences between the different taxa. For this reason we have considered all the members of clade A, including the former *Germanomeryx* and *Sinomeryx*, as belonging to the genus *Ampelomeryx*, because we find this taxonomic decision more congruent with respect to the distribution of character states in our topology, also giving a good and robust diagnosis of the genus. We have included the palaeomerycid from Sansan (‘*Palaeomeryx’ magnus*) into the genus *Triceromeryx*. This form shares with *T*. *pachecoi* a long proximo-lateral tubercle in the radius and the presence of a distal notch in the dorso-lateral border of the distal trochlea of the astragalus (node E). Its frontal appendages are of basal node C, cylindrical type [7]. The occipital appendage of ‘*Palaeomeryx’ magnus* remains unknown, however our topology rejects the close relationship of this form to *Ampelomeryx* (contrary to [7]). Thus, the most coherent approach is to include the fossils from Sansan in the genus *Triceromeryx* as *T*. *magnus*.

**Fig 11:**
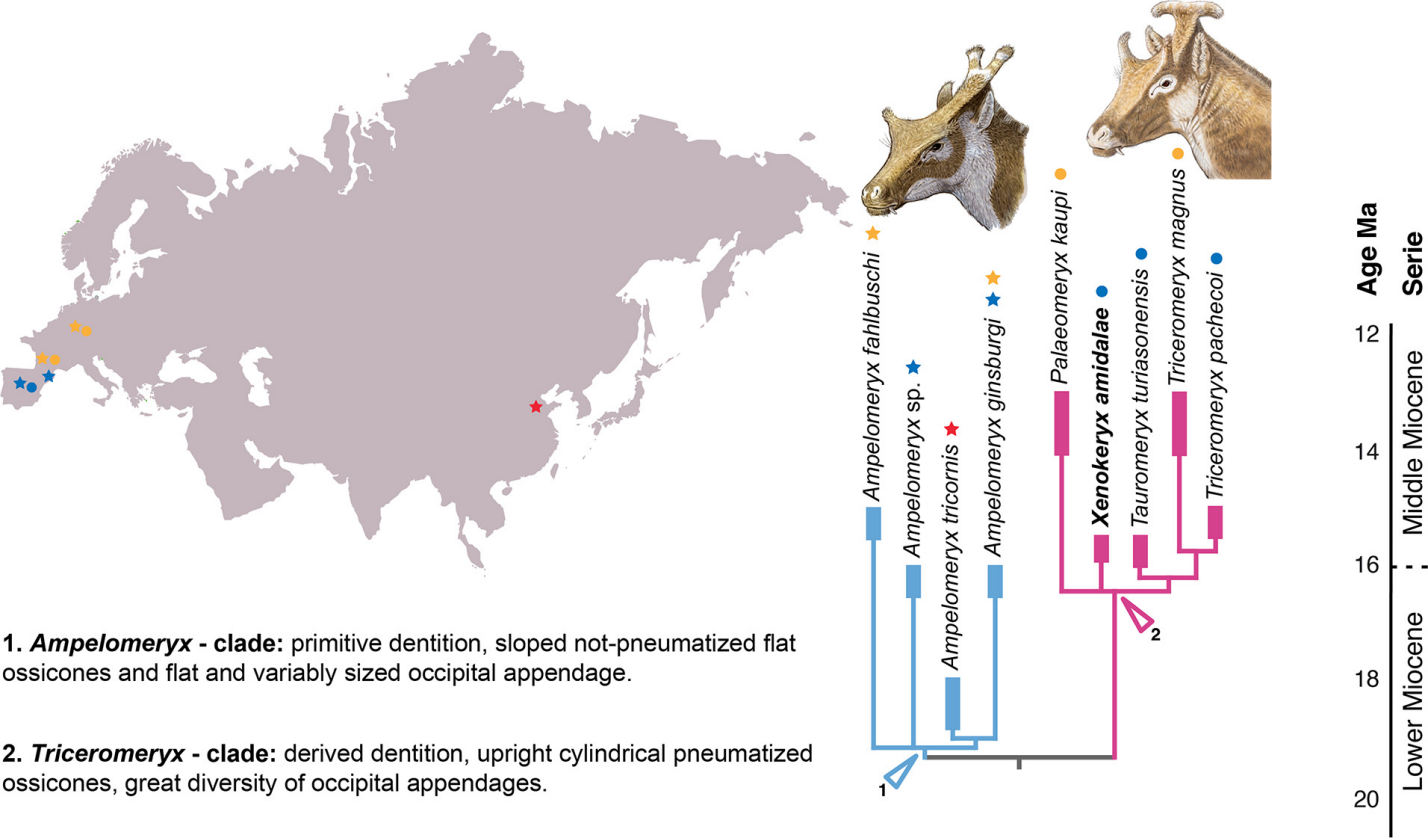
Evolution of palaeomerycids. Summary scheme showing a calibrated phylogeny of palaeomerycids (based on the corresponding MPT) with special emphasis on the main morphological traits of each basal clade (*Ampelomeryx*-clade and *Triceromeryx*-clade represented by reconstructions of *Ampelomeryx* and *Xenokeryx*) including their biogeographic distribution. Illustrations by IMS.

The *Ampelomeryx*-clade apparently had a more widespread paleobiogeographic distribution than the *Triceromeryx*-clade (Fig 11), the members of which have been mostly described from Europe and the Iberian Peninsula. However, there are two possible exceptions to this. Qiu & Qiu [16] cite *Tauromeryx*-like occipital appendages from Xiejahe (China), although they do not figure them. Also, Bohlin [20] described the species *Triceromeryx tsaidamensis* from the late Miocene of Tossun-Nor (China) on the basis of a single ossicone that he considered similar to those of *T*. *pachecoi*. Actually this specimen lacks the typical bumps of the ossicones from La Hidroeléctrica, only sharing with them the rounded tip. It is more similar to the Portuguese specimen figured by Antunes [73], but it is certainly akin to the ossicones of the *Triceromeryx*-clade. Thus, it is very plausible that members of this lineage, albeit abundant and diverse in the occidental part of Eurasia, were also present in Asia.

## Final remarks

Ruminants are the most abundant and diverse group of large terrestrial mammals. Since Oligocene times, ruminants have formed a major proportion of the world’s large herbivores, both in terms of diversity and biomass. However, the phylogenetic affinities of some ruminant clades, some with unique morphologies, and especially those without extant representatives, remains problematic [66]. Ruminants are widely distributed [74] and habitat sensitive [75], and thus eco-morphologically diverse. Reconstructing their evolutionary history, full of parallel morphological adaptations, and its link to changing environmental conditions can provide an indicator of major shifts in terrestrial ecosystems through time. To this end, placing problematic fossil taxa such as the paleomerycids in the ruminant tree is a fundamental task.

Our work may have important implications for the evolution of key features (e.g. cranial appendages). For example, our results indicate that ruminant lineages may have undergone major morphological adaptations in the late Oligocene or early Miocene (between 27 and 20 Ma), when some basal splits among pecorans took place. This timing significantly predates that obtained by a literal interpretation of the fossil record [76], predicting an earlier onset of the environmental conditions traditionally associated with this morphological diversity.

## Conclusions

We here present a new (albeit limited) phylogenetic analysis of the pecoran ruminants, with an emphasis on fossil forms and morphology, but also incorporating molecular data. A new palaeomerycid here described, *Xenokeryx amidalae* gen. et sp. nov. from the middle Miocene of Spain, helps to reinterpret and understand the morphological evolution and phylogenetic relationships of the group. Despite their apparent external similarities, Eurasian palaeomerycids are not related with North American dromomerycids. Instead, they belong in the clade that also contains the giraffes besides several extinct groups. We name this clade the Giraffomorpha. Among giraffomorphs, the early Miocene African pecoran *Propalaeoryx* is the closest sister group to palaeomerycids. On the other hand, dromomerycids are very closely related to cervids.

There are two main lineages of palaeomerycids. One of them, the *Ampelomeryx*-clade, is characterized by a well-developed *Palaeomeryx*-fold and several other dental derived characters (although they retain a relatively primitive dentition), sloped not pneumatized flat ossicones and flattish and variably sized occipital appendage. The other one, the *Triceromeryx*-clade, is characterized by its more derived dentition, upright cylindrical pneumatized ossicones, and a great diversity of occipital appendages.

This study focused mainly on the systematics of several extinct clades (palaeomerycids, dromomerycids and their respective allies). Future ruminant research will benefit from total-evidence phylogenetic methods (e.g. Bayesian tip-dating analysis used here) for combining fossil and living taxa, morphological and molecular datasets, and fossil ages. The inclusion of more living and fossil lineages in larger datasets will be decisive to further testing our findings and conclusions.

## Supporting Information

S1 File Matrix 1, Palaeomerycidae. (Simplified Nexus file).
(NEX)

S2 File Matrix 2, Palaeomerycidae within the Pecora, morphological dataset. (Simplified Nexus file).
(NEX)

S3 File Matrix 2, Palaeomerycidae within the Pecora, DNA dataset. (Simplified Nexus file).
(NEX)

S1 Table Measurements (cranial, dental, postcranial) of *Xenokeryx amidalae* gen. et sp. nov. from La Retama. (xlsx file).
(XLSX)

S1 Text Measurements (cranial, dental, postcranial) of *Xenokeryx amidalae* gen. et sp. nov. from La Retama. Description of the measurements. (docx file).
(DOCX)

S2 Text Character list 1, Palaeomerycidae. (docx file).
(DOC)

S3 Text Character list 2, Palaeomerycidae within the Pecora. (docx file).
(DOC)

## Abbreviations

AMNH: American Museum of Natural History, New York, USA
ICP: Institut Català de Paleontologia, Barcelona, Spain
MNCN-CSIC: Museo Nacional de Ciencias Naturales-CSIC, Madrid, Spain
MNHN: Musèum national d’Histoire naturelle, Paris, France
OTU: Operational Taxonomic Unit
MP: Maximum Parsimony
TBR: Tree Bisection Reconnection
