# Supplementary material for: Systematics and Evolution of the Miocene Three-Horned Palaeomerycid Ruminants (Mammalia, Cetartiodactyla)
Source: PLoS One. 2015 Dec 2;10(12):e0143034. doi: 10.1371/journal.pone.0143034 (PMC4668073; doi:10.1371/journal.pone.0143034)
Supplement: S1 Text — Description of the measurements. (docx file). (DOCX) [file pone.0143034.s005.docx]

**Postcranial Measurements.**

**Abbreviations: APD (anteroposterior diameter); DL (dorsal length);FAPD (functional anteroposterior diameter); FTD (functional transversal diameter); FL (functional length); H (height); TL (total length); TD (transversal diameter).**

*Scapula*.

1. Minimum neck APD.

2. Distal TD.

3. Distal APD.

4. APD glenoid cavity.

*Long bones (humerus, radius, ulna, femur, tibia).*

1. TL.

2. Proximal TD.

3. Proximal APD.

4. Distal TD.

5. Distal APD.

6. Minimum diaphysis TD.

7. Minimum diaphysis APD.

8. Humerus FL (from the distal articulation to the articular condyle).

9. Humeral trochlea TD.

10. Humeral trochlea APD.

11. Radius FL (from the distal articulation to the medial border of the proximal articulation).

12. Ulnar DL (DL of the tuber olecrani).

13. Ulnar tuber olecrani APD.

14. Ulnar anconeus process APD.

15. Ulnar tuber olecrani minimum APD.

16. Metacarpal III-IV magnotrapezoid facet TD.

17. Metacarpal III-IV unciform facet TD.

18. Metapodial supraarticular TD.

19. Femur FL.

20. Femur caput femoris TD.

21. Femur caput femoris APD.

22. Femur distal medial APD (APD of the medial border of the distal epiphysis).

23. Femur distal lateral APD (APD of the lateral border of the distal epiphysis).

24. Femur patellar trochlea TD.

25. FL tibia (from the proximo-medial condyle to the starting of the distal cochlea).

26. Distal FTD tibia (FTD of the distal cochlea).

27. Distal FAPD tibia (FAPD of the distal cochlea).

28. Metatarsal III-IV medial length (total length of the metatarsal III-IV from the proximo-medial border to the distal articulation).

*Carpal bones.*

1. Anterior H.

2. Unciform maximum H.

3. Pyramidal maximum H.

4. Pisiform posterior H.

5. Maximum APD.

6. Proximal TD.

7. Distal TD.

8. Semilunar palmar TD (TD of the palmar border measured in proximal view).

9. Magnotrapezoid anterior TD (TD measured in the dorsal part of the bone).

10. Magnotrapezoid posterior TD (TD measured in the palmar part of the bone).

11. Unciform semilunar facet TD (measured in the palmar border).

12. Unciform pyramidal facet TD (measured in the palmar border).

*Patella*.

1. Total H.

2. Maximum TD.

3. Maximum APD.

*Malleolar*.

1. Maximum H.

2. Posterior H.

3. Maximum TD.

4. Maximum APD.

Tarsal bones.

1. Calcaneum sustentaculum TL (L of the corpus measured up to the sustentaculum).

2. Calcaneum tuber calcis TD.

3. Calcaneum tuber calcis APD.

4. Calcaneum maximum TD including sustentaculum.

5. Calcaneum articular APD (APD from the anterior facet for the malleolar to the plantar border of the calcaneum.

6. Astragalus lateral TL.

7. Astragalus medial TL.

8. Astragalus lateral APD.

9. Astragalus medial APD.

10. Astragalus distal TD.

11. Cubonavicular anterior H (measured in the cuboid).

12. Cubonavicular anterior H (measured in the navicular).

13. Cubonavicular anterior total H.

14. Cubonavicular posterior total H.

15. Cubonavicular articular TD (TD of the proximal articulation).

16. Cubonavicular maximum TD of the facet for the ectomesocuneiform.

17. Cubonavicular maximum APD of the facet for the ectomesocuneiform.

18. Ectomesocuneiform maximum H.

Phalanges.

1. First and second phalanx TL.

2. Third phalanx TL (plantar border).

3. Third phalanx TL (dorsal border).

4. Minimum TD diaphysis.

5. Minimum APD diaphysis.

6. Proximal TD.

7. Proximal APD.

8. Distal TD.

9. Distal APD.

10. Second and third phalanx FAPD.
