# Supplementary material for: Systematics and Evolution of the Miocene Three-Horned Palaeomerycid Ruminants (Mammalia, Cetartiodactyla)
Source: PLoS One. 2015 Dec 2;10(12):e0143034. doi: 10.1371/journal.pone.0143034 (PMC4668073; doi:10.1371/journal.pone.0143034)
Supplement: S2 Text — (docx file). (DOC) [file pone.0143034.s006.doc]

**Palaeomerycidae character list.**

1. General morphology of the ossicone. 0, flattish; 1, cylindrical.

2. Extension of the nuchal crest into the shaft of the occipital appendage. 0, extended, forming a longitudinal ridge; 1, not extended.

3. Extension ‘wing’ in the frontal ossicones. 0, present, anterior to the ossicone; 1, present, posterior to the ossicone; 2, absent.

4.Morphology of the ossicone tip. 0, rounded and rugose, giraffe-like; 1, pointed.

5. Ornamentation in the ossicones. 0, large and individualized bumps, more or less abundant and concentrated in the posterior face of the ossicone; 1, more or less smooth surface with the presence of an occasional isolated bump; 2, more or less smooth surface, no bumps.

6. Pneumatization of the ossicone. 0, not pneumatized ossicone; 1, presence of pneumatization in the ossicone base.

7. Pedicle in the occipital appendage. 0, present; 1, absent or nearly absent.

8. Anteroposterior width of the occipital appendage. 0, broad appendage, elliptic to sub-cylindrical; 1, clearly narrow appendage, anteroposteriorly flat.

9. Morphology of the occipital appendage tip. 0, Y-shaped, narrow; 1, Y-shaped, broad, with short conical branches; 2, T-shaped; 3, Y-shaped with flat branches of variable length.

10. Length of the occipital appendage. 0, very long, almost as long as the skull; 1, medium length; 2, very short.

11. Posterior groove in the occipital appendage. 0, well-marked and triangular (tip pointing upwards); 1, poorly marked and triangular (tip pointing downwards); 2, not present.

12. Longitudinal ‘crests’ in the posterior face of the occipital appendage. 0. not present; 1, very well developed, forming two well marked longitudinal rods; 2, very reduced, with the shape of faint longitudinal crests.

13. Development of the nuchal tubercles. 0, flattish; 1, rounded and developed; 2, very developed, comparatively massive.

14. Morphology of the nuchal extension. 0, elongated and large; 1, short and triangular.

15. Morphology of the labial cone in the P4. 0, winged; 1, simple.

16. Presence of frontal supraorbital projections as ‘eyebrows’ in the base of the ossicones. 0, absence; 1, presence.

17. Morphology of the terminal end of the post-metacristid. 0, single; 1, bifurcated.

18. Relative morphology of p3/p4. 0, both teeth are similar in morphology and the p3 is also similar to the p4 in size; 1, p4 is bigger and more triangular than the p3.

19. Development of the labial groove in the p4. 0, faint and not reaching the base of the p4; 1, well-marked and reaching the base of the p4.

20. Morphology of the posterolabial conid in the p4. 0, elongated and narrow; 1, well-developed, triangular and large.

21. Mesolingual conid in the p4. 0, small and rounded; 1, well-developed and more triangular.

22. Morphology of the hypoconulid in the m3. 0, triangular and distally oriented; 1, elongated and in buccal position.

23. Orientation of the third lobe in the m3. 0, buccal; 1, central.

24. Development of the *Palaeomeryx*-fold. 0, short or almost absent; 1, well-developed.

25. Origin of the *Palaeomeryx*-fold. 0, protoconid; 1, post-protocristid.

26. General morphology of the magnotrapezoid. 0, high and transversally narrow; 1, low and transversally wide.

27. Development of the proximo-lateral tubercle in the radius. 0, short; 1, long.

28. Ulna fused distally with the radius. 0, not fused; 1, fused.

29. Development of the palmar extension of the facet for the semilunate in the radius. 0, short; 1, long, almost reaching the palmar end of the facet for the scaphoid.

30. Morphology of the dorsal distolateral border of the distal trochlea in the astragalus. 0, presence of a notch; 1, straight, with no notch.

31. Extension of the distal articulation facet of the first phalanx into the flexor area of the phalanx. 0, extended, with presence of triangular expansions; 1, not extended, with a straight flexor border.

32. Discrete tubercle for ligamentous attachment in the dorso-proximal part of the first phalanx. 0, not present; 1, located in the external side; 2, located in the interdigital side.
