# Supplementary material for: Systematics and Evolution of the Miocene Three-Horned Palaeomerycid Ruminants (Mammalia, Cetartiodactyla)
Source: PLoS One. 2015 Dec 2;10(12):e0143034. doi: 10.1371/journal.pone.0143034 (PMC4668073; doi:10.1371/journal.pone.0143034)
Supplement: S3 Text — (docx file). (DOC) [file pone.0143034.s007.doc]

*Character list*

1. Condition of the tympanohyal vagina: 0, without lateral enclosing; 1, reduced enclosing, affecting only the proximal third part of the tympanohyal; 2, complete enclosing, the tympanohyal vagina is not visible in lateral view.

2. Condition of the tympanic bulla. 0, not inflated, without posterior expansion; 1, laterally inflated, with moderated posterior expansion; 2, laterally inflated, with well developed posterior expansion.

3. Position of the tympanohyal vagina in the tympanic bulla. 0, caudal; 1, central or sub-central.

4. Separation between the processus styloideus and processus paroccipitalis. 0, without separation; 1, slightly separated by a thin bony sheet; 2, well separated by the intrusion of the tympanic bulla.

5. Contact between the tympanic bulla and the basioccipital. 0, no contact; 1, contact.

6. Lacrimal orifices. 0, one orifice; 1, two orifices, being clearly one above the other even if the upper one is more or less inside the lacrimal; 2, two orifices, almost aligned.

7. Location of the lacrimal orifices. 0, middle part of the anterior margin of the orbit; 1, dorsal part of the anterior margin of the orbit.

8. Condition of the optic foramina. 0, separated; 1, merged in a single foramen.

9. Retroarticular process. 0, nearly absent; 1, present with no expansions; 2, anteroposteriorly wide with well developed posterior expansions; 3, absent, with only a very robust lateral expansion; 4, developed with very large and triangular lateral expansion.

10. Contact between the postglenoid process and the external acoustic meatus. 0, no contact; 1, contact.

11. Morphology of the postglenoid foramen. 0, small; 1, large and laterally open; 2, large and laterally closed due to the expansion of the tympanic bulla; 3, small and laterally open.

12. Enclosing of the temporal canal. 0, open temporal canal; 1, laterally enclosed temporal canal due to the contact between the postglenoid process and the ectotympanic bone.

13. Cranial appendages. 0, absent; 1, antlers: non-permanent apophyseal appendages with hormonal regulation; 2, ossicones: epiphyseal perennial appendages that fuse to the frontals late in ontogeny; 3, horns: epiphyseal perennial appendages that fuse to the frontals early in ontogeny and have a perennial horn covering; 4, pronghorn-like appendages; 5, probable apophyseal appendages, perennial, branched by sprouting and probably with no hormonal regulation (Azanza et al. 2003); 6, probably apophyseal appendages (Bubenik, 1990), non-branched; 7, probably apophyseal, horizontal appendage formed by a X-shaped frame almost parallel to the skull with a ‘butterfly’ flatter expansion developed between the branches (in males).

14. Etmoidal vacuity. 0, absent; 1, developed without interrupting the nasal-lacrimal contact; 2, developed and interrupting the nasal-lacrimal contact.

15. Lacrimal fossa. 0, absent; 1, present, located in front of the lacrimal orifice(s); 2, present, located dorsally to the lacrimal orifice(s).

16. Occipital/mastoid relationship. 0, well developed mastoid bone that forms part of the nuchal crest along the occipital bone; 1, small mastoid that does not form part of the nuchal crest.

17. Opening in the border of the mastoid, between the mastoid and the occipital. 0, absence; 1, presence of circular opening; 2, presence of slit-like elliptical opening; 3, presence of slit-like opening included within a large fossa.

18. Occipital appendage. 0, absence; 1, presence of forked occipital appendage formed by the elongation of the nuchal plane; 2, presence of non-forked occipital appendage of supraoccipital origin with no nuchal modification involved.

19. Location of the parieto-squamosal suture. 0, located in the middle of the upper and lower borders of the temporal fossa; 1, located near the inferior border of the temporal fossa.

20. Condition of the lateral margin of the infratemporal fossa. 0, poorly developed; 1, well-marked.

21. Depth of the infratemporal fossa. 0, shallow; 1, deep.

22. Separation between the foramen ovale and the infratemporal fossa. 0, separated by a low and sharp small crest; 1, separated by a well-developed crest; 2, not directly adjacent.

23. Contact between the acoustic tube and the mastoid bone. 0, no contact; 1, contact.

24. Nuchal fossa. 0, absent; 1, present.

25. Presence of a deep rectilinear groove between the caudal part of the temporal fossa and the nuchal plane. 0, absence; 1, presence.

26. Hipselodont molars. 0, absence; 1, presence.

27. Morphology of the P4. 0, triangular in shape, with a centrally located and well developed lingual cone; 1, with more or less individualized cones; 2, semicircular in shape and selenodont, with flat and poorly individualized cones.

28. Morphology of the P3. 0, elongated with an individualized central cone; 1, shortened with an individualized central cone; 2, semicircular in shape and selenodont.

29. Morphology of the P2. 0, elongated, with lingual cone poorly developed; 1, well developed lingual cone present; 2, shortened.

30. p1. 0, present; 1, absent.

31. p2. 0, present; 1, absent.

32. Arrangement of p4 elements. 0, lineal; 1, transversal.

33. Arrangement of p3 elements. 0, lineal; 1, transversal.

34. Buccal sulcus in the p4. 0, absent; 1, present.

35. Morphology of the p4. 0, without lingual elements; 1, the mesolingual conid joins the cristid obliqua through the posterolingual cristid; 2, as in 1 and with very developed anterolingual cristid that encloses totally or almost totally the anterior valley; 3, the mesolingual conid joins directly the cristid obliqua; 4, absence of cristid obliqua, and the anterolingual cristid joins the anterior wing, whereas the posterolingual cristid is poorly developed and does not reach the posterior wing; 5, shortened p4 with all cuspids united into a continuous occlusal surface.

36. Morphology of the posterolingual conid of the p4. 0, elongated and narrow, belonging to the posterior wing; 1, large, broad and triangular-shaped, almost individualized; 2, elongated and fully individualized, with a shape similar to that of a lower molar cusp.

37. Condition of the p4 cusps. 0, well differentiated from their cristids; 1, not individualized.

38. Alineation of the lingual cusps of the lower molars. 0, not aligned; 1, completely or almost completely aligned.

39. Condition of the upper canines of males. 0, canines absent; 1, presence of robust and wide hypertrophied canines that develops vertically with a more or less pronounced distal curvature; 2, presence of long, slender and narrow saber-like canines showing a proximally concave and distally convex lateral surface.

40. Development of the postentocristid (connection hypoconid/entoconid). 0, not developed, or poorly developed, postentocristid; 1, developed entocristid, almost contacting the posthypocristid; 2, highly developed postentocristid that fuses with the posthypocrisitd, distally closing the lower molars; 3, fusion related with hypsodonty /hypselodonty.

41. Development of the postprotocrista. 0, short postprotocrista; 1, expanded postprotocrista.

42. Morphology of the lower canines. 0, plain crown; 1, bi-lobed crown.

43. Morphology of the third lobe of the m3. 0, monocuspidate; 1, bicuspidate, with presence of well developed post-entoconulidcristid; ; 2, bicuspidate with very small entoconulid; 3, bicuspidate and extremely well developed third-lobe almost as large as the anterior lobes; 4, bicuspidate in fully hypsodont / selenodont dentition, with non-individualized cuspids.

44. Development of the lingual cingulid. 0, present; 1, absent.

45. Caprine-fold. 0, absent; 1, present.

46. *Palaeomeryx*-fold. 0, present; 1, absent.

47. Presence of ‘M-structure’ in the lower molars. 0, presence; 1, absence.

48. General morphology of the lower molar cusps. 0, pyramid-like in shape; 1, more or less flattened, with narrower lingual ribs.

49. Vertical development of the humeral capitulum. 0, little developed; 1, developed; 2, very developed.

50. Morphology of the capitular facet in the radius. 0, short and wide, without dorsal development; 1, elongated and wide; 2, short and wide, without palmar development; 3, similar to state 2 but with a conspicuous triangular dorsal process.

51. Morphology of the facet for the semilunate in the radius. 0, absence of lateral notch; 1, presence of lateral notch.

52. Condition of the distal articular facets of the semilunate. 0, subequal; 1, the facet for the unciform is larger; 2, the facet for the magnotrapezoid is larger.

53. Condition of the canal for the digital common artery in the metatarsal III-IV. 0, ‘tragulid-type’, superficially placed but not in an equally fused metatarsals as in the Pecora, with poorly developed groove; 1, ‘moschid-type’, superficially located, but not as superficial as in ‘3’; 2, ‘cervid-type’, very deeply located, in the mid-shaft axis of the metatarsal; 3, ‘bovid-type’, extremely superficially located, almost without groove.

54. Location of the plantar column of the metatarsal III-IV. 0, central; 1, lateral.

55. Condition of the plantar facet for the navicular-cuboid in the metatarsal III-IV. 0, sloped; 1, horizontal.

56. Metatarsal plantar tuberosity. 0, absent; 1, well developed; 2, small.

57. Supraarticular fossetes in the metatarsal III-IV. 0, absent; 1, present.

58. Condition of the metatarsal sulcus. 0, distally open; 1, distally closed.

59. Condition of the distolateral facet for the astragalus in the calcaneum. 0, convergent with the dorsal border of the corpus; 1, parallel with the dorsal border of the corpus.

60. Development of the astragalar distal trochlea. 0, developed (distal half of the astragalus roughly equal to the proximal half); 1, short (distal half of the astragalus much shorter than the proximal half).

61. Development of the proximo-plantomedial process of the navicular-cuboid. 0, little developed; 1, developed.

62. Morphology of the proximo-plantomedial process of the navicular-cuboid. 0, without any crest; 1, presence of a round-shaped crest that develops in the distal part of the plantomedial process; 2, presence of a well developed crest that does not reach the proximal region of the plantomedial process; 3, presence of a highly developed crest that extends along the entire plantomedial process.

63. Development of a central canal in the plantar side of the navicular-cuboid, lateral to the proximo-plantomedial process. 0, navicular-cuboid without canal; 1, presence of a little developed canal; 2, presence of a well-developed canal that sports a marked concavity.

64. Morphology of the lateral border of the dorsodistal process of the tibia. 0, presence of a well marked step; 1, smooth and sloped.

65. Morphology of the astragalus. 0, non-parallel sides; 1, parallel sides.

66. Development of the metapodial keels. 0, not developed into the extensor region of the articular facet; 1, developed into the extensor region of the articular facet.

67. Laterally-oriented expansion of the nuchal crest. 0, absent; 1, present.
